# Antioxidant, Anti-Inflammatory, and Immunomodulatory Properties of Tea—The Positive Impact of Tea Consumption on Patients with Autoimmune Diabetes

**DOI:** 10.3390/nu13113972

**Published:** 2021-11-07

**Authors:** Anna Winiarska-Mieczan, Ewa Tomaszewska, Karolina Jachimowicz

**Affiliations:** 1 Institute of Animal Nutrition and Bromatology, University of Life Sciences in Lublin, Akademicka St. 13, 20-950 Lublin, Poland; karolina.jachimowicz@up.lublin.pl; 2 Department of Animal Physiology, Faculty of Veterinary Medicine, University of Life Sciences in Lublin, Akademicka St. 12, 20-950 Lublin, Poland

**Keywords:** autoimmune diabetes, tea, polyphenols, antioxidant, anti-inflammatory, immunomodulatory

## Abstract

The physiological markers of autoimmune diabetes include functional disorders of the antioxidative system as well as progressing inflammation and the presence of autoantibodies. Even though people with type 1 diabetes show genetic predispositions facilitating the onset of the disease, it is believed that dietary factors can stimulate the initiation and progression of the disease. This paper analyses the possibility of using tea as an element of diet therapy in the treatment of type 1 diabetes. Based on information available in literature covering the last 10 years, the impact of regular tea consumption or diet supplements containing tea polyphenols on the oxidative status as well as inflammatory and autoimmune response of the organism was analyzed. Studies conducted on laboratory animals, human patients, and in vitro revealed positive effects of the consumption of tea or polyphenols isolated therefrom on the diabetic body. Few reports available in the literature pertain to the impact of tea on organisms affected by type 1 diabetes as most (over 85%) have focused on cases of type 2 diabetes. It has been concluded that by introducing tea into the diet, it is possible to alleviate some of the consequences of oxidative stress and inflammation, thus limiting their destructive impact on the patients’ organisms, consequently improving their quality of life, regardless of the type of diabetes. Furthermore, elimination of inflammation should reduce the incidence of immune response. One should consider more widespread promotion of tea consumption by individuals genetically predisposed to diabetes, especially considering the drink’s low price, easy availability, overall benefits to human health, and above all, the fact that it can be safely used over extended periods of time, regardless of the patient’s age.

## 1. Introduction

Diabetes entails a combination of metabolic, autoimmune, and genetic disorders leading to hyperglycemia [1]. The hyperglycemia is due to either the impairment of insulin production, the lowering of cellular sensitivity to insulin, or a combination of the two factors. Chronic hyperglycemia damages, impairs the function, or leads to the failure of a variety of organs, in particular, the eyes (it can result in blindness), nerves, kidneys, heart (infraction), and blood vessels (stroke) [2]. It can also be the cause of gangrene (potentially necessitating amputation) and neuropathies, especially in adult patients [2]. Maintaining glycemia within normal physiological levels significantly limits the emergence and progression of typical diabetes complications in the form of microangiopathies, however, ensuring correct metabolic equilibrium in diabetics is not sufficient to fully prevent the development of such microangiopathies [3].

The etiological classification of diabetes proposed by the World Health Organization (WHO) distinguishes between three major types of the disease: type 1 (insulin-dependent, resulting from the non-secretion of insulin by pancreatic β cells due to the destruction of such cells); type 2 (non-insulin-dependent, resulting from lowered sensitivity of target tissues to the effects of insulin); and pregnancy diabetes [2]. One of the most common consequences of uncontrolled diabetes is chronic hyperglycemia. Such conditions are conducive to autooxidation of glucose and the formation of reactive oxygen species (ROS), which in turn leads to micro- and macrovascular dysfunction as well as polyneuropathies caused by the organism’s endogenic antioxidative defenses [4]. The resulting oxidative stress triggers fragmentation or structural deformation of lipids, denaturation of proteins, disorders in the mechanisms of DNA replication, and deformation of cellular organelles, and consequently entire cells [5]. As such, uncontrolled diabetes can lead to multisystem failures related to microvascular endpoints including retinopathy, nephropathy, and neuropathy as well as macrovascular endpoints including coronary artery disease, stroke, and peripheral artery disease [6].

The etiology of type 1 diabetes has yet to be fully understood. The disease usually emerges in children and adolescents. The sufferers’ blood serum shows the presence of pancreatic β islet-cell antibodies and glutamic acid decarboxylase as well as anti-insulin antibodies, and antibodies active against tyrosine phosphatase, which trigger the gradual destruction of cells producing insulin by immune T cells [7]. The role of modifiable factors causing type 2 diabetes is somewhat better known, which renders prophylaxis a more viable goal in terms of public health [8]. Type 2 diabetes is responsible for a vast majority (approx. 90%) of all diabetes cases and is observed primarily in older patients whose blood shows no presence of the antibodies [9]. In some patients clinically diagnosed with type 2 diabetes, antibodies active against pancreatic β islet-cells are present, which indicates a case of latent autoimmune diabetes in adults (LADA), which is considerably more difficult to diagnose [10]. It has been recently proposed that LADA should be defined as “slowly progressive insulin-dependent type 1 diabetes” (SPIDDM), as the patients whose blood reveals the presence of glutamine acid decarboxylase antibodies and/or pancreatic β islet-cell antibodies are initially not dependent on insulin and do not experience ketose or ketoacidosis [11]. Due to the shortage of large, multi-center clinical studies, it is difficult to definitively establish the incidence of LADA, but it has been estimated, however, that it affects around 12% of all cases of diabetes in adults [12]. Given the fact that the autoimmune process in LADA is less aggressive than in cases of classic type 1 diabetes, studies are now being undertaken with a view to determining the possibility of therapeutic interventions that could reduce the progression of β cell failure [11,13].

The primary course of diabetes treatment entails pharmacotherapy aimed at lowering blood glucose levels. The pharmacological treatment of diabetes is long-term, often life-long, which exacerbates the risk of adverse reactions and harmful impact of the drugs on the patients’ overall health. The most common side effects include brain damage, erythema, stomach and gastrointestinal disorders, excessive body mass, metallic aftertaste in the mouth, heart failure, and vitamin B12 deficiencies [14]. All of the above suggest the need for less invasive, but also more effective methods. Given the fact that the physiological markers of diabetes include disorders of the antioxidative system as well as progressing inflammation and the presence of specific antibodies, the primary form of adjunctive treatment that should accompany pharmacotherapy should entail food rich in substances capable of aiding the organism in overcoming these types of disorders. As suggested in the literature, one type of such substances are polyphenols that show a range of pharmacological and therapeutic properties, primarily in terms of their antioxidative and anti-inflammatory activity. The immunomodulatory properties of polyphenols may, in turn, be useful in alleviating the symptoms of autoimmunological disorders. Polyphenols are capable of activating intracellular pathways (e.g., the arachidonic acid-dependent pathway, the nuclear transcription factor (NF-κB), mitogen-activated protein kinases (MAPK), phosphatidylinositol 3-kinase/B protein kinase signaling pathway (PI3K/Akt) as well as stimulating epigenetic modulations that regulate the organism’s immune response [15]. Food rich in polyphenols is easily accessible and can be used chronically, regardless of the patient’s age. Tea, one of the world’s most popular drinks, second only to water, is certainly among the possible options [16]. Tea contains a range of substances with antioxidative, anti-inflammatory, and immunomodulatory properties including tannic acid, catechins (e.g., epigallocatechin-3-gallate EGCG present in green tea), theaflavins, and thearubigins present in black tea as well as quercetin [17,18]. Overall, polyphenols correspond to between 25 and 35% of total dry leaf mass [18]. Their content is the highest in white tea, followed by the green, black, and red varieties [19].

The paper analyses the possibility of using tea as an element of diet therapy in cases of type 1 (autoimmune) diabetes. Based on information available from worldwide literature published in the last 10 years, the impact of regular tea consumption on the oxidative status, emergence of inflammation, and autoimmune response was analyzed.

## 2. Materials and Methods

**Figure 1:**
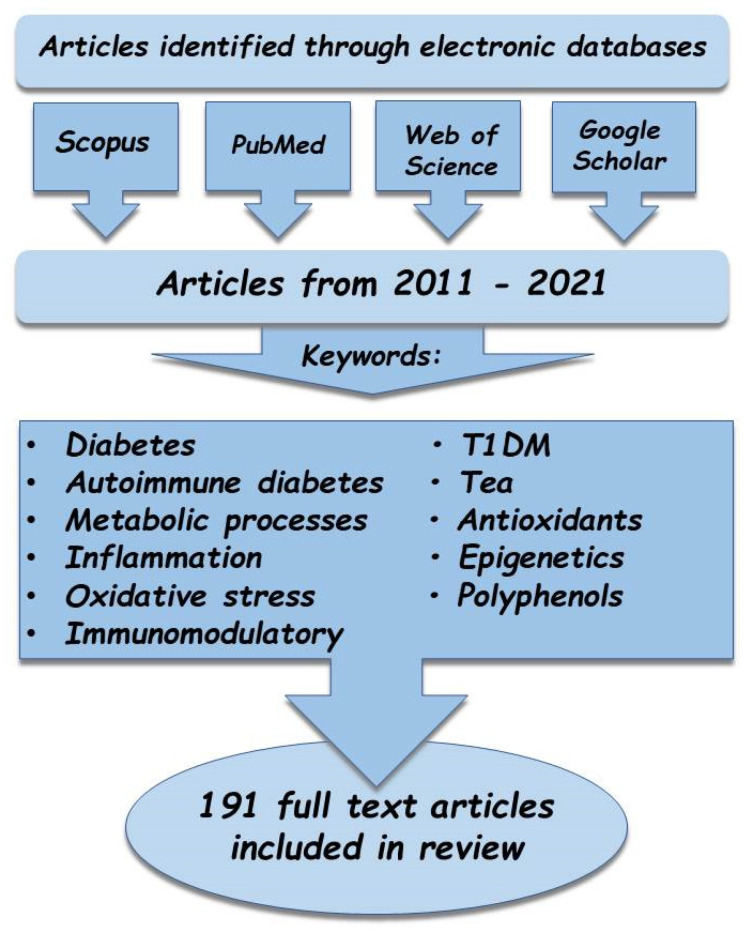
Research strategy employed in the review of the available literature.

The analysis of information available in the global scientific literature was conducted in August 2021 using the following databases: Scopus, PubMed, Web of Science, and Google Scholar. The databases were searched for both joint and separate instances of the keywords: “diabetes,” “autoimmune diabetes”, “T1DM”, “tea”, “metabolic processes”, “inflammation”, “oxidative stress”, “antioxidants”, “immunomodulation”, “epigenetics”, and “polyphenols”, in Polish and English (Figure 1). Based on an analysis of the titles and synopses, articles unrelated to the substantive criteria were excluded, and the remaining research and review papers were analyzed in greater depth to identify the most pertinent publications. Bibliographies were also reviewed in all the selected articles to identify other potentially viable texts. The search was narrowed to papers published between 2011 and 2021. Ultimately, a total of 2546 publications were reviewed, of which 191 were used: 116 research reports and 75 reviews.

## 3. Pathogenesis of Autoimmune Diabetes

Type 1 diabetes is an autoimmune disease mediated by T cells [15]. In nearly all patients diagnosed with type 1 diabetes before the age of five, the presence insulin-specific antibodies has been reported, which suggests a significant role of peptides originating from insulin in the pathogenesis of the disease [7]. Autoantibodies are a marker facilitating the diagnosis of autoimmune diabetes. Histological analyses of the pancreas in autoimmune diabetes patients revealed infiltrations of immune cells, macrophages, dendric cells, NK (natural killer) cells as well as antibodies reacting to pancreatic islets and T cells reacting to Langerhans islets [20]. Conditioning immune cells have the ability to generate immune memory when coming in contact with an antigen, and when the contact is repeated, they induce immune response [21]. The immune response triggers the production of proinflammatory cytokines that promote phagocytosis, autophagy, and interferon activity, which in turn lead to cell death [3,22]. There are several etiopathogenetic models for type 1 diabetes: (1) the autoimmune process is triggered by autoreactive T cells and antibodies emerging after pancreatic β islet-cells are damaged due to primary initiating factors; (2) it is caused by an upset food antigen tolerance due to functional disorders in the immune system of gastrointestinal mucosae; (3) due to the similarity between exogenous antigens and β islet-cell antigens, the pancreatic immune response is directed against β cells; (4) coexistence of β cell susceptibility to apoptosis, autoimmune response against β cells, and insulin resistance; and (5) genetic predispositions: the presence of class II DR and DQ genes of the human leukocyte antigen HLA on chromosome 6 (DDM1) [3,23]. The pathological mechanisms involved in the progression of type 1 diabetes include DNA methylation, modification of histones and microRNA as well as molecular mimicry, acting through the regulation of gene expression [24].

LADA diabetes is a form of autoimmune diabetes that affects adult patients and is characterized by the presence of circulating β cell antibodies [25]. It entails a chronic autoimmune process that results in the destruction of pancreatic islets [11]. The dynamic of that process is slower than in the case of type 1 diabetes, but in time, the insulin secretion disorders are gradually exacerbated, which triggers more severe symptoms of the disease [13]. LADA is diagnosed when (1) the minimum age of the patient developing diabetes is 30 years; (2) the presence of islet antibodies is detected in the organism; and (3) absence of inulin was not observed for at least six months after diagnosis [13]. As LADA patients are initially non-insulin-dependent and diagnosis is based solely on the presence of islet antibodies, the identification of LADA can be difficult in a clinical context [11]. The genetic variants in the HLA complex in LADA patients is the same as in type 1 diabetes patients [26].

In type 1 diabetes, class II genes in the HLA system located on chromosome 6p21.3 are the most significant to the genetic predisposition of sufferers. The polymorphism alleles in these genes are responsible for approx. 50% of the genetic predisposition for diabetes. Class II DR3 and DR4 haplotypes in the HLA system play a particularly important role as at least one of the same is found in 90% children and young with type 1 diabetes [22]. A protective role is attributed to the DR2 haplotype [27]. Approx. 15% of the genetic predisposition for developing type 1 diabetes is attributed to the insulin promotor (insulin-linked variable number of tandem repeats INS-VNTR, chromosome 1p5,511p15), the cytotoxic T-Lymphocyte Antigen-4 receptor (CTLA-4, chromosome 2q33), protein tyrosine phosphatase N22 (PTPN2), and the immune signaling regulator (PTPN22, chromosome 1p13) as well as other genes [28,29]. INS-VNTR polymorphism is responsible for the expression of the insulin gene not only in the pancreas, but also thymus [29]. Mutation or polymorphism of the CTLA-4 gene leads to an uncontrolled proliferation response, which can be the cause of autoimmune diseases including type 1 diabetes [30]. In the case of LADA, the frequencies of DR3 and DR4 haplotypes are similar to those observed in type 1 diabetes patients [31]. Some data suggest that in individuals with LADA, DR3 and DR4 haplotypes occur more often than in the general population [32], which indicates a genetic predisposition for islet autoimmunization. Moreover, a comparison between LADA and type 1 diabetes revealed no directional differences in terms of the frequency of class II alleles emergence, which suggests that both diseases have the same underlying genetic cause [32]. It has been demonstrated that in approx. 60% of LADA patients, polymorphisms of the CTLA-4 gene, in particular the G CTLA-4 alleles, is observed, and the likelihood of the disease increases if diabetes is present in the family [33].

## 4. Metabolic Disorders in Diabetes

Under the conditions of imbalance between the processes of oxidation and antioxidation resulting from the failure of the antioxidative system, cells begin to produce excessive ROS, triggering oxidative stress [17]. This leads to an inflammatory response as well as triggers NF-κB protein-dependent transcription of genes for various inflammatory factors [34]. Inflammation is, among other things, a defensive mechanism allowing the cells of an organism to protect themselves against pathogens and damaging factors (e.g., autoimmune reactions) [35]. In such a case, phagocytes are activated (monocytes, neutrophils, eosinophils), which release proinflammatory cytokines at the site of damage (e.g., interleukins IL-1, IL-6, IL-8, tumor necrosis factor α TNF-α, interferon γ, IFN-γ). It cannot be excluded that environmental factors play an important role in inducing autoimmune responses already in the fetal period [36].

### 4.1. Oxidative Stress

Concentrated glucose solutions can alter the properties of many cells, above all endothelial cells, neutrophils, monocytes, and platelets [37,38]. Hyperglycemia intensifies glucose metabolism in endothelial cells, granulocytes, monocytes, and platelets, which is accompanied by the increased production of reactive oxygen species, leading to disturbance of the intracellular oxidoreductive balance and oxidative stress [39,40,41]. In endothelial cells, glucose metabolism along the polyol pathway is intensified, which results in a decreased ratio of the reduced from of NADPH (nicotinamide adenine dinucleotide phosphate) to its oxidized form (NADP+) and increased the ratio of the reduced form of NADH (nicotinamide adenine dinucleotide) to the oxidized form thereof (NAD+) [42]. NADH is excessively produced due to hyperglycemia in glycolytic pathways and Krebs cycle as well as the activation of the polyol pathway, whereas NAD+ is reduced due to overactivation of poly-ADP-ribose polymerase that uses the compound as a substrate [43]. NAD+ is also used by sirtuins as a substrate in catalyzing the reaction of protein deacetylation. Inhibition of the synthesis of regenerative NAD+ enzymes (e.g., lactate dehydrogenase in erythrocytes) and complex I (the first enzyme of the respiratory chain) in mitochondria, it may also contribute to the accumulation of NADH and NAD+ deficiency. Disorders in terms of NADH and NAD+ oxidation are also responsible for the overproduction of reactive oxygen species [44,45,46]. Whereas the lowered intracellular content of NADPH reduces its availability (e.g., for glutathione, i.e., one of the body’s primary antioxidative systems), which additionally exacerbates oxidative stress [44,47]. Under oxidative stress, glucose metabolism must be relocated from the glycolytic to the pentose phosphate pathway, which facilitates the production of NADPH necessary for maintaining the reduced states of glutathione and thioredoxin with the participation of glyceraldehyde-3-phosphate dehydrogenase (GAPDH) [48].

The conditions of hyperglycemia also intensify the non-enzymatic glycosylation of proteins with the accompanying oxidation of glucose as well as inactivation of SOD—a very biologically active sweeper of free radicals [49,50,51].

Oxidative stress constitutes the primary factor connecting hyperglycemia with intensified protein glycation, activation of protein kinase C, formation of glycosaminoglycans, and activation of NF-κB responsible, among other things, for the development of an inflammatory reaction [52,53,54]. All of the above disorders lead to a modification of cell functions, which alters their autocrine and paracrine properties, and can even cause their death [55,56]. Moreover, oxidative stress triggers a cytotoxic effect, which may cause structural DNA changes and consequently disturb the proliferation and regeneration of the epithelium [57]. Under oxidative stress, mitochondria are damaged, leading to their dysfunction [35,58]. The concentration of free Ca^+2^ in the cytosol is increased, inducing cell activation [59]. Oxidation of protein –SH groups to –S–S–disulfide bridges under the influence of oxidative stress reduces the compensative efficiency of antioxidative mechanisms and inactivates many enzymes [60]. Oxidation of polyunsaturated fatty acids by toxic oxygen derivatives produces lipid peroxides, which also show oxidative properties [49]. Through indirect participation in radical reactions, lipid peroxides cause the production of fatty acids, generating highly reactive and toxic lipid radicals [61]. Peroxidation of cellular membrane lipids alters their functional and antigenic properties and modifies receptor expression. As endothelial permeability increases in the conditions of hyperglycemia, peroxidized lipids can infiltrate beyond the vascular bed, while long-term oxidation of polyunsaturated fatty acids and peroxide fragmentation with a view to producing aldehydes eventually leads to the loss of the integrity of cytomembranes by altering their liquidity [62,63]. During intensive lipid peroxidation, the extent of oxidative cell damage exceeds their reparative capacity, which induces apoptosis or necrosis, leading to cell death [62]. Meanwhile, under physiological or subtoxic conditions, cells survive due to endogenic antioxidants, triggering an adaptive response to oxidative stress.

Mitochondria are the main source of oxidative stress in diabetes [49,64]. During oxidative metabolism, mitochondria reduce oxygen to H_2_O, while any excess oxygen is converted into reactive oxygen species O•, and then into peroxynitrite ONOO−, hydroxy radical OH•, and hydrogen peroxide H_2_O_2_ [46,48,65]. Additionally, the lipids of mitochondrial membranes are susceptible to damage. Peroxidation of mitochondrial phospholipids leads to structural changes and may consequently disturb the organization of the lipid layer by influencing its liquidity and permeability; it can also lead to depolarization of the mitochondrial membrane, reduction in ATP production, and intensified production of ROS [64]. The overproduction of ROS in mitochondria, particularly the superoxide anion radical O_2_•^–^, inhibits the activity of glyceraldehyde-3-phosphate dehydrogenase, which participates in glycolysis, consequently leading to the accumulation of glucose and its incorporation into alternative metabolic pathways [64,66]. ROS production is regulated through the enzymatic and non-enzymatic antioxidative system. The most important parameters describing oxidative stress in the organism are enzymes: superoxide dismutase (SOD), catalase (CAT), and glutathione peroxidase (GPX) as well as non-enzymatic antioxidants present in cells, mainly glutathione (GSH) [17]. Oxidative stress plays an important role in the onset of diabetes complications, both in the microvascular and cardiovascular system [50]. One of the reasons behind the functional disturbance of antioxidative systems is the overproduction of O_2_•^–^, generated in the course of the one-electron reduction in O_2_ and H_2_O_2_ as a by-product of tissue oxidation in mitochondria [46].

Studies conducted on male C57BL/6 mice in whom hyperglycemia was induced with streptozotocin showed an increase in MDA and a decrease in total antioxidant capacity (TAC), SOD total, SOD2 (Mn-dependent SOD), and the GSH/GSSG (glutathione disulfide) ratio in the endothelium [67]. Cohort studies conducted in a group of 382 diabetic children reported reduced serum GPX, SOD, and CAT levels in comparison with healthy children [68]. Similarly, in a study conducted by Varvarovská et al. [69,70], reduced levels of antioxidant parameters (SOD, GSH, TAC) and increased MDA were detected in 50 children with type 1 diabetes compared to healthy children. Concurrently, it was observed in this study that the oxidatively damaged DNA in children with diabetes was not significantly altered compared to that of the healthy children, which probably indicates an increased rate of DNA repair, probably as a response to the constantly elevated oxidative stress. Patients with type 1 diabetes often have elevated ketone levels, and ketosis increases lipid peroxidation and lowers GSH levels in human cells, as demonstrated in in vitro studies [71,72]. Abnormalities in the expression of enzymes protecting against oxidative damage may aggravate various types of oxidative damage. In analyses of peripheral blood mononuclear cells collected from 26 patients with type 1 diabetes and 10 healthy individuals, Hodgkinson et al. [73] demonstrated an inhibitory effect of hyperglycemia on the expression of CAT, CuZnSOD, GPX, and MnSOD. Babizhayev et al. [54] proposed that variants within genes encoding the antioxidant enzymes: catalase (CAT), glutathione peroxidase 1 (GPX1), and glutathione transferase (GST) may contribute to the genetic susceptibility to diabetic neuropathy in type 1 diabetes. The researchers examined 466 patients with type 1 diabetes for up to three years. Their study demonstrated a protective role of the −262T CAT allele and the “+” GSTM1 allele against the rapid development of oxidative stress in type 1 diabetes related to increased levels of CAT, GSH, and GST in patients with these alleles.

### 4.2. Inflammation

Under the conditions of hyperglycemia, pathological metabolic processes are triggered in the organism including the polyol pathway and hexosamine pathways or non-enzymatic protein glycation. Hyperglycemia activates the apoptosis of β cells due to the activity of cytokines produced by the subpopulation of Th1 lymphocytes: INFγ, IL-2, and 1IL-18; interleukins produced by macrophages: IL-1, IL-6, IL-8, and IL-12; and TNFα. It is believed that cytokines released by the subpopulation of Th2 lymphocytes: IL-4, IL-5, IL-10, and IL-13 act protectively, inhibiting the activity of Th1 lymphocytes, which is particularly apparent in early stages of the disease [74,75]. In response to inflammation, blood serum proteins are produced that act as mediators of the ongoing inflammatory process, mostly high sensitivity c-reactive protein hsCRP and interleukins [75]. CRP is produced primarily in hepatocytes in response to other mediators of inflammation, especially interleukins and TNF-α [76]. TNF-α, together with IL-2 and IL-6, stimulates the proliferation and differentiation of B and T cells, facilitates the functions of autoreactive CD4 and CD8 lymphocytes, and influences the function and number of Treg lymphocytes by inhibiting their suppressor activity. TNF-α aids the activity of macrophages and neutrophils, stimulates the production of other proinflammatory cytokines, induces the synthesis of reactive oxygen species, and peroxidation of lipids [77]. The biological activity of TNF-α depends on the interactions with a specific receptor as well as the number of receptors present on the surfaces of target cells. So far, two types of TNF-α receptors have been discovered: TNFRI (55kDa) and TNFRII (75kDa) [78]. Interleukins play an important role in immune and inflammatory response. Interleukin IL-1β induces the proliferation and differentiation of B cells, intensifies their chemotaxis, and together with IL-6 and TGF-β (transforming growth factor), stimulates the differentiation of lymphocytes toward Th17, which is responsible for quick inflammatory response and migration of neutrophils [77]. Moreover, IL-1β stimulates the production of proinflammatory cytokines (IL-2, IL-6, TNF-α, IFN-γ) and contributes to the destruction of pancreatic β cells [79]. IL-6 stimulates the proliferation and differentiation of B cells into plasmacytes producing antibodies, and T cells into cytotoxic Tc lymphocytes; it also activates the synthesis of acute-phase proteins in the liver, particularly CRP [76,80]. A study conducted in a group of 125 patients with type 1 diabetes revealed that proinflammatory cytokines IL-6, TNF-α, and IFN-γ as well as anti-inflammatory cytokine IL-10 showed a mutual positive correlation, which may suggest their supplementary activity in the context of the emergence and progression of vascular complications in diabetics [81]. A study conducted in Poland in a group of 71 children (aged 7–17) with type 1 diabetes demonstrated that from the first years of the disease, elevated concentrations of inflammation markers (hsCRP, IL-6, IL-1) could be observed compared to healthy children [75]. In turn, a Turkish study conducted among children with type 1 diabetes confirmed the activation of a systemic inflammatory process already in the early stages of the disease, which may indicate ongoing destruction of β cells, whereas as the disease continues to progress, the levels of IL-1β, IL-2, IL-6, and TNF-α confirm continuous activation of proinflammatory factors, even in late stages of diabetes [79]. In rats intragastrically administered with pure fructose dosed at 0, 2.6, 5.3, or 10.5 g/kg/day for 20 weeks, an increase in the serum concentration of proinflammatory cytokines (IL-6, TNF-α, and MIP-2) increased, while the level of anti-inflammatory cytokine IL-10 was significantly reduced [82].

Oxidative stress triggered by ROS not only leads to inflammatory response, but also induces NF-κB protein-dependent transcription of genes for a variety of inflammatory factors [34]. The activation of NF-κB due to oxidative stress results in higher production of cytokines, increased expression of adhesive molecules as well as intensified cell apoptosis [83]. This facilitates the formation of a specific inflammatory reaction within the vascular wall, whose pathogenic role in damaging the vascular wall, particularly in the context of atherosclerosis, has been demonstrated in vitro and in studies conducted on laboratory animals [37,84,85]. Increased ROS production by polynuclear neutrophils at the site of inflammation causes endothelial dysfunction and tissue damage, which in turn leads to the opening of mesothelial connections and facilitates migration of inflammatory cells through the endothelial barrier [86]. Migrating inflammatory cells aid the elimination of pathogens and foreign particles, but also cause tissue damage [86].

### 4.3. Autoimmune Disorders

The initiation of a specific immune response depends on the recognition of the foreign nature of an antigen as well as the conditions under which the given antigen is presented to immunocompetent cells: if the conditions are interpreted by the organism as pathological, they will trigger an immune response regardless of whether the antigen is the organism’s own or foreign [87]. In the case of auto-aggression, the signal inducing the immune response may originate from an inflammation accompanying the release of antigens due to damage [88]. The immunological markers of type 1 diabetes include antibodies active against pancreatic β cells: Islet-cell antibodies (ICA), insulin autoantibodies (IAA), anti-glutamine acid decarboxylase (anti-GAD), anti-zinc transporter protein 8 (anti-ZnT8), and anti-tyrosine phosphatase antibodies (anti-IA2) [89]. They are detectable many months before clinical symptoms of diabetes, signify the humoral immune response against Langerhans’s pancreatic β islet-cells, and are considered to be markers of pancreatic cell destruction. The presence of one of the said antibodies has been confirmed in 95% of affected patients, hence they can serve as effective early markers given their sustained presence in the patients’ blood serum for a number of years preceding the onset of diabetes [90]. A study conducted in a group of 78 Moroccan children with type 1 diabetes, all under the age of 16, revealed the presence of anti-GAD antibodies in approx. 63% of them, anti-IA2 in 77%, and simultaneously both of the same in 53% of the subjects, notably more commonly in girls [89]. In an Iranian study, the presence of antibodies was confirmed in over 80% of children and adolescents with type 1 diabetes, primarily ICA and Anti-GAD [91]. Notably, also in this study, the presence of antibodies was more commonly detected in girls. In approximately 5–10% patients diagnosed with type 2 diabetes, the markers of β cell autoimmunization also emerge—such cases are qualified as LADA [92]. Patients with autoimmune diabetes also show the presence of antibodies related to the coincidence of other autoimmune diseases: 20% have anti-thyroid peroxidase (anti-TPO) and/or anti-thyroglobulin (anti-TPO) antibodies, 11% have antibodies evidencing the presence of coeliac disease (antigliadin anti-DGP, anti- tissue transglutaminase TG, anti-endomysial EMA), 2% have anti-adrenal antibodies (a marker of Addison disease), 1% have antibodies active against parietal cells (markers of autoimmune gastric mucositis) [93]. Moreover, studies conducted among LADA patients (*n* = 70) and type 2 diabetes patients (*n* = 69) revealed that LADA patients more commonly had antibodies active against thyroid antigens (anti-TPO, anti-TG) as well as against tissue transglutaminase of IgA class (anti-tTG, coeliac disease marker), indicative of subclinical hypothyroidism [94].

In the case of diabetes, once pancreatic islets are damaged by initiating factors, antigen-presenting cells (APCs) activate helper CD4+ T cells, activated in the course of diabetogenesis by peptides present in the β f insulin [95]. Active CD4+ T cells, via the lymphokines they produce, induce apoptosis/necrosis of pancreatic β cells, causing infiltration of mononuclear cells into pancreatic islets [96]. The process of inducing autoantigens on the surface of β cells is facilitated by internal (IFN-γ, TNF-α, and IL-1β, free radicals) or external factors (toxins, viruses), but the autoimmune process itself is initiated in the β cells of Langerhans islets [96]. Insulin released by β cells may be an autoantigen initiating the immunological cascade together with, for example, T cells, as a consequence of which type 1 diabetes emerges—as demonstrated in a study on NOD mice [97].

## 5. Antioxidative, Anti-Inflammatory, and Immunomodulatory Properties of Tea

### 5.1. Antioxidative Properties

Due to its high content of polyphenols (mainly EGCG, quercetin, theaflavin, thearubigin, tannic acid), in other words, substances with strong antioxidative properties, tea can in fact be classified as functional food. Phenolic compounds show antioxidative properties thanks to their ability to: (1) sweep ROS; (2) limit the production of ROS by inhibiting the activity of oxidative enzymes and chelating trace elements; and (3) increasing the activity of endogenic antioxidants [3,5,17]. The particularly strong antioxidative activity of EGCG is due to the compound’s chemical structure, which includes as many as eight –OH groups [17]. Catechins act primarily by transferring H+ ions, but also fairly likely through mechanisms that directly or indirectly regulate the expression of enzymatic antioxidants [98]. The antioxidative properties of quercetin are due to its ability to donate an electron or hydrogen atom, which allows it to neutralize singlet oxygen (1O_2_), O_2_•^–^, OH•, LOO•, NO, and ONOO– [17,99]. This, in turn, is responsible for quercetin’s ability to neutralize ROS by inhibiting the activity of enzymes participating in their formation (e.g., oxidases) and enzymes using NADPH as a coenzyme [17]. The highest antioxidative capacity, reflecting the highest content of total polyphenols, characterizes green and white tea varieties [19,100].

Numerous studies have demonstrated increased activity of superoxide dismutase (SOD), CAT, GST, and GPX as well as overall increased glutathione (GSH) content in the tissues of animals receiving tea extracts or polyphenols isolated therefrom, which indicates an increased capacity of antioxidative mechanisms due to the supply of exogenous antioxidants, which facilitates the balance of redox reactions and prevents oxidative stress (Table 1 and Table 2). Studies on a system simulating the process of oxidation in the human organism revealed that green and black tea extracts were able to strongly inhibit the formation of linolic acid peroxides [101]. Similar results were reported by Korir et al. [102] in a study on mice. After 12 weeks of administering black, green, white, and red tea extracts to Wistar rats poisoned with prooxidative, toxic metals, an increase in SOD, CAT, and GPX activity in the animals’ organs was observed [19], where the positive results were similar to those observed for tannic acid [103]. Kombucha tea administered to rats poisoned with cadmium chloride improved the antioxidative capacity of the organism [104,105]. Quercetin administered to rats poisoned with cadmium improved the oxidative status by increasing the activity of SOD, CAT, and GPX, and lowering that of lipid peroxidation (LPO), malondialdehyde (MDA), and H_2_O_2_ [106,107]. A study by Simos et al. [98] conducted on rats demonstrated a decrease in MDA levels and increase in SOD in urine after intragastric administration of catechin and epicatechin. An improvement in terms of the antioxidative parameters (SOD, CAT, GPX, MDA, LPO) in the blood serum of mice was reported after the administration of polyphenols isolated from green tea (50, 100, or 200 mg/kg) [108]. Administration of EGCG to rats exposed to electromagnetic radiation led to an improvement in antioxidative parameters (SOD, CAT, GSH) and decrease in MDA; notably, the authors observed better effectiveness when EGCG was used simultaneously with the stressor rather than after the stress period [109]. Tea polyphenols significantly alleviated damage to the ileum due to Salmonella typhimurium in C57BL/6 mice, while also causing a decrease in inflammation and oxidative stress markers by improving the overall antioxidative status of the organism [110]. In studies utilizing human colorectal cancer cell lines (Volo-205), it was reported that lipid peroxidation was reduced after the application of tea polyphenols [111], whereas in human colorectal cancer cells HCT-116 and SW-480, a decrease in terms of the markers of oxidative stress and cell proliferation was reported [112]. In an in vitro study on rats subjected to stress, the use of theaflavin improved the recorded oxidative stress biomarkers [113].

### 5.2. Anti-Inflammatory and Immunomodulatory Properties

As confirmed in in vitro and in vivo studies conducted to date on polyphenols and extracts rich in the same, the compounds showed considerable anti-inflammatory properties (Table 1). The primary effects that polyphenol have on the course of inflammation stems from their ability to inhibit the synthesis of proinflammatory cytokines, INF-γ, TNF-α, and chemokines in various types of cells [131]. Moreover, polyphenols show anti-inflammatory activity on many levels, mainly by inhibiting NF-κB, regulating mitogen-activated protein kinase (MAPK), inducible nitric oxide synthase (iNOS), and arachidonic acid, cyclooxygenase-2 (COX-2), and lipoxygenase (LOX) as well as lowering ROS synthesis relative to reactive nitrogen species [132,133]. An important target for the activity of polyphenolic compounds is NF-κB, which plays an important role in immunological and inflammatory processes [134]. By inducing proliferation and stimulating angiogenesis in cells, NF-κB controls the expression of proinflammatory cytokines and chemokines (IL-1α, IL-1β, IL-2, IL-6, IL-8, TNF-α), COX-2 as well as some growth factors and apoptosis regulators [134]. Hence, factors that limit the activation of NF-κB may also prevent the expression of cytokines, and consequently block inflammatory response. EGCG inhibits the activation of NF-κB and MAPK as well as the expression of IFNγ, TNF-α, and IL-1β, while also stimulating the innate expression of immunity-related genes (e.g., TNF-α, MAPK, NOS) and inhibiting apoptosis [135]. EGCG may also inhibit the infiltration of inflammatory and pro-inflammatory leukocytes IL-8, while studies on mice revealed that it can lower the expression of pro-inflammatory factors: NF-κB and IL-6 [135,136,137]. Thichanpiang and Wongprasert [118] demonstrated that EGCG shows anti-inflammatory effects on human retinal pigmented epithelial cells ARPE-19, partially as a suppressor of TNF-α signaling, and that the inhibitive effects occur along the NF-κB pathway. EGCG prevented the production of the plasminogen activator inhibitor-1 (PAI-1) in the cells of human umbilical vein endothelium via TNF-α and reduced the phosphorylation of regulated protein kinases ERK1/2 [138]. PAI-1 is involved in numerous physiological processes, but also many pathologies (e.g., polymorphisms –765 4G/5G and –844 A>G are a predisposition for elevated glucose and insulin levels in the blood serum). PAI-1 is considered to be an acute phase protein; its release is stimulated by proinflammatory factors, primarily IL-1 and NF-κB [139]. EGCG minimizes damage to endothelial cells, reducing the production of IL-6 and TNF-α by inhibiting the activity of AP-1, a protein activating transcription factors 1 NF-kB [140,141]. It also inhibits the production of CRP induced by macrophage angiotensin II (AII) and IL-6 by limiting the production of free oxygen radicals [142]. Consumption of green tea extract by obese individuals, combined with moderate physical activity, facilitates an increase in anti-inflammatory adiponectin and hsCRP, but does not significantly influence the levels of IL-6 and TNF-α [143]. A decrease in CRP levels was reported in smokers drinking four cups of green tea a day [142], similar results were also observed in individuals with hypertension [144]. Chen et al. [145] demonstrated the anti-inflammatory properties of a tea-flower extract in acute and immunological inflammations triggered by croton oil and carrageenan as well as Propionibacterium acnes and liposaccharide. In the cited study, a decrease in the levels of NO, TNF-α, and IL-1β was observed. In the in vitro studies conducted by Chatterjee et al. [146] with the use of water, black, and green tea extracts revealed the inhibition of egg albumin denaturation, which demonstrates tea’s anti-inflammatory properties. In the cited study, it was concluded that green tea is more active than black, probably due to the higher content of flavonoids.

T and B cells are key components of the adaptive immune system [147]. Immune cells are equipped with various types of receptors including ones dedicated to polyphenols that recognize polyphenols and allow the cells to trap them. Afterward, polyphenols activate signaling pathways and initiate specific immune responses of the organism; they can also induce epigenetic changes in cells [148]. Tea polyphenols and their derivatives act by stimulating numerous signaling pathways, as demonstrated in in vivo and in vitro studies [135]. Polyphenols have an immunomodulatory influence on macrophages, increase the proliferation of B cells, T cells, and suppress the activity of type 1 helper T cells (Th1), Th2, Th17, and Th9 cells and show immunomodulatory activity against allergic reactions and autoimmune disease by inhibiting the autoimmunological proliferation of T cells [147]. Zhou et al. [136] demonstrated an improved ration of CD3+CD4+ T to CD3+CD8+ T lymphocytes, which increased in C57BL/6J mice with induced Parkinson’s disease after oral administration of EGCG. Similarly beneficial results were reported in a study on mice with autoimmune arthritis, wherein decreased levels of proinflammatory cytokines and lower degrees of T cell proliferation were observed after administering EGCG [149]. When administered to mice with autoimmune encephalomyelitis, EGCG reduced the clinical symptoms of the disease as well as the pathological immune response [150]. An inhibitive impact of EGCG on the release of inflammatory cytokines was reported in activated human primary T cells, most likely due to inactivation of Ap-1 [151]. In a study on piglets, it was demonstrated that tea polyphenols promote the proliferation of immune cells, T cell activation, increased concentration of CD4+ T lymphocytes, increased values of the CD4+/CD8+ ratio as well as improvement in T cell transformation (LTT) [152]. Studies conducted on shrimp infected with the white spot syndrome and Vibrio alginolyticus bacteria revealed a positive impact of EGCG on the expression of pro-immune genes such as IMD, proPO, QM, myosin, Rho, Rab7, p53, TNF-α, MAPK, and NOS [153].

## 6. Impact of Tea on Organisms with Autoimmune Diabetes—A Review

**Figure 2:**
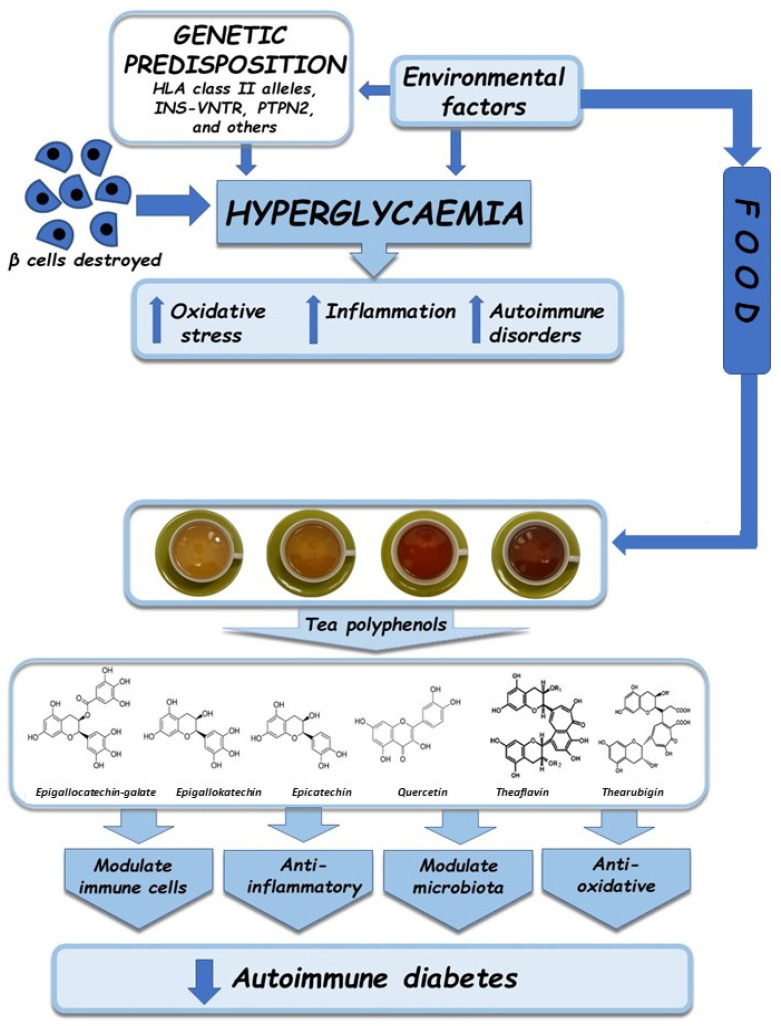
The factors that influence the development of autoimmune diabetes and the therapeutic significance of tea polyphenols.

Consumption of exogenous polyphenols may play an important role in gaining or maintaining immunity by way of interrupting the synthesis of proinflammatory cytokines, thus regulating immune cells gene expression [151,153]. Studies on laboratory animals, humans as well as in vitro have confirmed the positive effects of consuming tea or polyphenols isolated therefrom on organisms with autoimmune diabetes (Table 1 and Table 2). The protective effects of tea in the context of diabetes and related complications are due to a number of mechanisms related to: (1) strengthening the effects of insulin; (2) reducing insulin resistance; (3) activating the insulin signaling pathway; (4) protecting β islet-cells; and (5) eliminating free radicals and alleviating inflammation [154,155,156] (Figure 2). EGCG, whose highest content is found in green tea, shows multidirectional anti-hypoglycemic properties: it inhibits the production of glucose in the liver, promotes phosphorylation of the insulin receptor and insulin receptor substrate-1, controls glucogenesis by inhibiting the expression of carboxy phosphoenolpyruvate carboxy-kinase and glucose 6-phosphatase genes, regulates the expression of genes contributing to the pathways involved in insulin signal transfer and glucose uptake, alleviates β cell damage caused by cytokines, and improves insulin sensitivity [155]. On the other hand, theaflavin, whose highest content is found in black tea, inhibits the activity of α-glucosidase, which lowers glucose production in the intestine [157]. It was observed that rats with glucose intolerance receiving flavan-3-ols showed improvement in terms of pancreatic islet functions, which suggests that the compounds may act as cellular signaling molecules modulating the insulin output [158]. One of the proposed mechanisms of catechin activity entails increased cellular production of ROS mediated by pro-oxidative EGCG, which leads to the activation of protein kinase by adenosine monophosphate, which in turn inhibits the expression of genes, enzymes, and transcription factors involved in adipogenesis and lipogenesis [159]. The effectiveness of epicatechins depends on their concentration in the organism: high levels of epicatechins can significantly reduce the production of ROS induced by H_2_O_2_ or by hyperglycemia in β cells [160]. The cited authors also observed that epicatechins, even at lower doses, are capable of restoring insulin secretion via the Ca^2+^/CaMKII pathway by activating GPR40 in pancreatic β cells. Komorita et al. [161] demonstrated, based on a study conducted in a group of 4923 Japanese patients with type 2 diabetes, that consumption of high amounts of green tea was corelated with lower mortality, which was related to the high supply of phenolic compounds, particularly EGCG. In another study, after analyzing the dietary habits of 40,530 Japanese subjects, it was found that the risk of death due to any cause was 15% lower for individuals consuming at least five cups of green tea a day, compared to those consuming less than one cup a day [162].

The literature provides a few study reports pertaining to the impact of tea on organisms with type 1 diabetes; the vast majority of publications (over 85%) have focused on type 2 diabetes only. However, oxidative stress and inflammation both constitute physiological markers common to both types of hyperglycemia. Type 1 diabetes is additionally characterized by autoimmune reactions, which are closely connected to the emergence of inflammation. Based on the available research, it can be concluded that a proper diet can alleviate the effects of oxidative stress and inflammation, regardless of the type of diabetes. Alleviation of inflammation can reduce the incidence of immune responses.

### 6.1. Antioxidative Activity

Administration of a white tea extract to rats with hyperglycemia triggered a significant increase in SOD, CAT, GPX, and GSC-Px levels as well as a decrease in MDA levels in the liver and blood serum, which indicates stimulation of the synthesis of exogenous antioxidative enzymes [126]. Similarly, in a study by Sharifzadeh et al. [127], the use of green tea extract in rats with induced diabetes revealed that the experimental factor lowered LPO and total thiol group content but did not influence the TAC level. In rats receiving fructose for eight weeks, partial inhibition of the CuZnSOD and GPx activity was reported. Prince et al. [114] explained the same by suggesting inhibition of the excessive superoxide anion production due to the simultaneous administration of (-)-epicatechin. It should be noted, however, that in this particular study, the measured parameters were similar to those obtained in the control group. However, in another study conducted under the same conditions by Calabró et al. [116], increased levels of antioxidative parameters were reported in rats receiving (-)-epicatechins compared to animals receiving a diet containing fructose dosed at 20 mg per 1 kg of body mass. A short-term exposure to oxidants increases the activity of endogenic antioxidants, which suggests activation of defensive mechanisms and adaptive cell response, but under the conditions of long-term oxidative stress, their activity is clearly lowered due to the expulsion of antioxidative metals from active enzyme centers [19,163]. Theaflavin administered to rats with induced diabetes lowered the levels of lipid peroxidation markers as well as other oxidative stress markers, while increasing the activity of antioxidative enzymes and exogenous non-enzymatic antioxidants [119,125,164]. During in vitro digestion of a water extract of matcha tea, polyphenols become more bioavailable and are characterized by higher antioxidative and antidiabetic activity when compared to sencha tea [165].

### 6.2. Anti-Inflammatory Activity

Studies conducted on isolated human coronary endothelial cells cultured on media containing elevated levels of glucose as well as research conducted on C57BL/6 mice, demonstrated the positive impact of (-)-epicatechin on the levels of glucose itself as well as markers related to the biogenesis of mitochondria through the activation of eNOS (endothelial nitric oxide synthase) under normal and simulated diabetic conditions [166]. The diet of Sprague Dawley rats receiving fructose in the form of 10% water solution for eight weeks was supplemented by the addition of (-)-epicatechin, which eliminated or alleviated the negative consequences of the high fructose intake, as evidenced by lowered levels of inflammatory factors (NF-κB, TNFα, iNOS, IL-6, nuclear/cytosolic p65 ratio) in the kidneys [114]. Administration of (-)-epicatechin (20 mg/kg bm) to rats receiving a 10% water solution of fructose prevented the activation of NF-κB and the increase in the activity of NADPH 4 (NOX4) oxidase in the renal cortex [116]. At the same time, the cited authors concluded that the absence of changes in the activity of TRL-4 (actively involved in NF-κB activation) in the renal cortex, both after the administration of fructose and (-)-epicatechin, suggests that only internal factors (e.g., antioxidants) can affect the activation of NF-κB. In a study by Mota et al. [123], it was reported that when Swiss mice injected with a solution containing 300 mg of carrageenan with a view to inducing inflammatory response, were administered orally or subcutaneously with an alcohol extract of green tea, the same inhibited the migration of inflammatory cells to the peritoneum. It has been demonstrated that EGCG inhibits the activity of the NF-κB factor, prevents the activation of the IκB kinase, and consequently limits expression of the genes regulated by the factor [167]. The strong anti-inflammatory (lowering of TNF-α, IL-1β, IL-6 levels) effects of pu-erh tea were shown in a study conducted on hyperlipidemic rats and cells with inflammatory lesions [168].

Studies suggest that EGCG can affect the strength of both innate and adaptive abilities of the immune system by influencing its regulation and increasing the number of regulator T cells [149]. As autoimmune disorders are closely related to inflammation (studies emphasize the role of T cells as the primary factors connecting inflammation and autoimmune pathology) [169], it can be assumed that as inflammation markers are lowered due to the consumption of tea or polyphenols isolated therefrom, the organism’s immune sensitivity is also decreased. It has been demonstrated that green tea can correct microbial dysbiosis, influencing the growth of bacteria contributing to inflammatory states by facilitating the development of beneficial bacteria, inhibiting the growth of harmful ones, or increasing the production of desirable metabolites such as short-chain fatty acids [170]. Short-chain fatty acids show anti-inflammatory and immunomodulatory properties [171]. Restoration of intestinal microflora is necessary in the context of reducing the intensity of inflammatory processes that stimulate autoimmune processes [172,173].

### 6.3. Immunomodulatory Activity

The available literature lacks information on the impact of tea consumption on the presence of antibodies active against pancreatic β cells in type 1 diabetes patients. However, patients with autoimmune diabetes often also show the presence of antibodies associated with other coexisting autoimmune diseases including hypothyroidism (20%), coeliac disease (11%), Addison’s disease (2%), and autoimmune gastric mucositis (1%): anti-TPO, anti-TG, anti-DGP, anti-TG, and anti-EMA [92,93]. Maintaining a correct diet by eliminating some foods and including others can limit the release of such antibodies if the comorbidity is confirmed, thus contributing to the overall improvement of the diabetes patient’s health. The positive effects of tea consumption on the health of patients with various autoimmune diseases have been confirmed in numerous studies [5,65,149,174,175].

The literature provides some information regarding the immunomodulatory properties of tea, particularly that of EGCG. EGCG shows the capacity to interact with and modulate the bioavailability of the primary immunomodulating 33-amino acid peptide originating from gluten, as demonstrated in in vitro studies [176]. The peptide, when not bound to a chelator (e.g., EGCG), after the deamination of glutamine into glutamine acid by way of tissue transglutaminase, binds with the antigen of the HLA-DQ2 or HLA-DQ molecule. The complex is subsequently presented to T CD4+ lymphocytes, whose activation is related to the production of cytokines such as IFN-γ, IL-2, -IL4, IL-10, and TNF-α, and consequently, the emergence of inflammation that leads to the atrophy of intestinal villi [174,176]. EGCG can control the expression of genes through epigenetic modification [177].

## 7. Perspectives and Conclusions—Can Nutrigenomics Be the Future?

Even though patients with type 1 diabetes are genetically predisposed for the disease, it is believed that environmental factors stimulate the onset and progression of the disease [178]. Epigenetic modifications, changes regulating the expression of genes, are also important [179]. Of the latter, DNA methylation in the regions of the promoted genes is the best understood change leading to gene inactivation, and the process is reversible in the reaction of demethylation [180]. DNA analyses allowed for the identification of 88 methylation sites in B cells including those influencing genes related to the pathogenesis of diabetes such as HLA and subunit β of the interleukin receptor 2 (IL-2Rβ), and in terms of the entire genome of human pancreatic islets, 383 potential methylation locations have been identified [179]. Studies indicate that micro-RNA can participate in the autoimmune damage to β cells, regulation of the synthesis and release of insulin, and consequently, the pathogenesis of type 1 diabetes [181].

Diet is an important factor influencing the course of type 1 diabetes and the emergence of related complications. As green tea and EGCG show pleiotropic activity, one might consider their possible application with a view to improving the quality of life of patients with inflammatory conditions. Epidemiological studies revealed that Chinese and Japanese populations, which traditionally consume large amounts of green tea, are among those with the lowest incidence of type 1 diabetes in the world [182,183]. This may be due both to the high antioxidative, anti-inflammatory, and immunomodulatory properties of tea polyphenols as well as their modulatory impact on human DNA. It is known that bioactive ingredients of food and diet supplements can alter molecular expression and/or genetic structure [184]. It is possible to modify one’s diet in such a way to improve one’s health and reduce the risk of many diseases. However, the effectiveness of nutrigenomics can be ensured only if we understand the interactions between a given nutrient and specific genes in the given organ or tissue. Only then will one be able to predict how an individual genetic system (DNA transcribed on mRNA, and then proteins) will respond to a specific nutrient [184]. Nutrients can modify the expression of genes involved in the organism’s immune response, either directly or through changes in intestinal microflora [179]. It has been demonstrated that EGCG reduces the level of expression of DNA damage-induced transcript-3 (Ddit-3), the marker of endoplasmic reticulum stress and its further signaling targets including Cdkn1a as well as protein phosphatase 1, regulatory subunit 15A (Ppp1r15a) [185]. The lowered expression of the mentioned markers facilitates better pancreatic function and lower insulin resistance as well as higher β cell vitality [184]. In type 1 diabetes, there is a deficiency of the insulin receptor substrate Irs-2, whereas EGCG stimulates higher expression of Irs-2 as well as B protein kinase (Akt) and O1 protein (Foxo1) [185]. Due to the modulation of the expression of CLL/lymphoma 2 from B cells (Bcl-2), EGCG protects β cells by producing insulin before the onset of cytotoxicity induced by proinflammatory cytokines [186].

Green tea and EGCG exert a positive health impact without significant side effects; nonetheless, caution should be exercised as under some circumstances (genetic conditions, medicines), the consumption of tea may in fact have adverse consequences, up to and including liver damage. Gallo et al. [182] went as far as to conclude that in certain specific cases, green tea may be a potential trigger for autoimmune hepatitis. The hepatotoxicity is most likely a result of enzymatic interactions (alcohol dehydrogenase, P450 cytochrome, mitochondrial enzyme) leading to cell damage and interference with the systems of biological response and metabolic reactions [187]. However, studies demonstrating hepatoprotective qualities of green tea are decidedly more common [19,188,189].

Type 1 diabetes develops only in 10–15% of individuals with the specific genotype predisposing them to the disease [190], which further confirms the key impact of environmental factors in the disease’s induction. One should consider more widespread promotion of tea consumption by individuals genetically predisposed for diabetes, especially considering the drink’s low price, easy availability, overall benefits to human health, and above all, the fact that it can be safely used over extended periods of time, regardless of the patient’s age.

## Figures and Tables

**Table 1:** Antioxidant and anti-inflammatory effects of tea polyphenols.

Polyphenols	Protective Effect		Design	Animals	References
	Antioxidant parameters	Inflammatory parameters			
(-)-epicatechin	↓ TBARS; ↓ SOD; ↓ GPX	↓ ratio nuclear/cytosolic p65; ↓TNF-α; ↓ iNOS	10% (*w*/*v*) fructose in the drinking water for 8 weeks; (-)-epicatechin (20 mg/kg body weight/day) in diet for 8 weeks	Male Sprague Dawley rats	[114]
(-)-epicatechin	↑ NOS; ↓ O_2_-;		10% (*w*/*v*) fructose in the drinking water for 8 weeks; (-)-epicatechin (20 mg/kg body weight/day) in diet for 8 weeks	Male Sprague Dawley rats	[115]
(-)-epicatechin	↑ NOS; ↑ SOD; ↑ GPX; ↓ CAT; ↓ TBARS		10% (*w*/*v*) fructose in the drinking water for 8 weeks; (-)-epicatechin (20 mg/kg body weight/day) in diet for 8 weeks	Male Sprague Dawley rats	[116]
(-)-epicatechin	↓ TBARS; ↓ SOD; ↑ NOS	↓ TNFα; ↓ iNOS; ↓ IL-6	10% (*w*/*v*) fructose in the drinking water for 8 weeks; (-)-epicatechin (20 mg/kg body weight/day) in diet for 8 weeks	Male Sprague Dawley rats	[117]
EGCG	↓ ROS;	↓ ICAM-1; ↓ NF-κB	Cells were pretreated with or without 100 µM EGCG for 1 h prior to exposure without or with 20 ng/mL of TNF- for 24 h	Human retinal pigment epithelial ARPE-19 cells	[118]
Theaflavin	↑ SOD; ↑ CAT; ↑ GSH; ↑ GST; ↓ TBARS; ↓ HP		100 mg/kg bw /day theaflavin administered orally to diabetic rats for 30 days	Male Wistar diabetic rats	[119]
EGCG	↓ MDA; ↓ TOS; ↑ thiols; ↑ CAT; ↑ TAC;		60 mg/100 g bw streptozotocin by intraperitoneal injection; 2.5 mg/100 g bw/day EGCG in saline solution or in liposomal form by intraperitoneal injection for 2 days	Male Wistar-Bratislava diabetic rats	[120]
EGCG	↑ SOD; ↓ ROS; ↓ RAGE mRNA;	↓ TNF-α; ↓ IL-6	25 mM glucose; 2.2 mM EGCG	Human embryonic kidney 293 (HEK293) cells	[121]
Catechin	↓ MDA; ↑ SOD; ↑ CAT; ↑ GST		Streptozocin by intraperitoneal injection; 40 or 80 mg/kg/day catechin by intraperitoneal injection for 4 weeks	Male diabetic Wistar rats	[122]

↓—decreased or inhibited concentration or activity compared to untreated group; ↑—increased concentration or activity compared to untreated group; EGCG—epigallocatechin-3-gallate; GPX—glutathione; SOD—superoxide dismutase; CAT—catalase; GSH—reduced glutathione; GST—glutathione-S-transferase; MDA—malondialdehyde; HP—hydroperoxides; TBARS—thiobarbituric acid reactive substance; iNOS—inducible nitric oxide synthase; NOS—nitric oxide synthase; ROS—reactive oxygen species; O_2_-—superoxide anion; TOS—total oxidative status; TAC—total antioxidant capacity; RAGE—receptor for advanced glycation end products; TNF-α—tumor necrosis factor α; IL-6—interleukin-6; ICAM-1—intercellular adhesion molecule 1; NF-κB—nuclear transcription factor.

**Table 2:** Antioxidant and anti-inflammatory effects of tea.

Polyphenols	Protective Effect		Design	Animals	References
	Antioxidant parameters	Inflammatory parameters			
Alcoholic extracts of green tea		↓ inflammatory cell migration in the peritoneum	0.07 or 0.14 g alcoholic extracts of green tea per kg by gavage or subcutaneously one hour before intraperitoneal injection of carrageenan (inflammation induction)	Male Swiss mice	[123]
Green tea extract	↑ TAS	↓ TNF-α; ↓ CRP	2 or 4 g extract of green tea per 1 kg of high-sodium-diet (35 g/kg) for 42 days	Male Wistar rats	[124]
Green tea extract	↑ GSH; ↑ SOD; ↑ CAT; ↑ GSH-Px; ↓ MDA		Green tea extract (1.5%, *w*/*v*) as a sole drinking source	Male Wistar diabetic rats	[125]
White tea extract	↑ SOD; ↑ CAT; ↑ GPX; ↑ GSH-Px; ↓ MDA		White tea extract (2%, *w*/*v*) as a sole drinking source	Male diabetic rats	[126]
Green tea extract	↓ LPO; ↓ total thiol groups		Green tea extract (3 mg/L) as a sole drinking source	Male diabetic Wistar rats	[127]
Green tea extract		↓ TNF-α; ↓ CRP; ↓ IL-6; ↓ NF-κB	Streptozocin by intraperitoneal injection; 300 mg green tea extract for 9 weeks	Male Sprague-Dawley rats	[128]
Green tea water extract		↓ TNF-α; ↑ IL-10	Streptozocin by intraperitoneal injection; green tea solution (7 g/L) ad libitum for 5, 30, 60 or 90 days	Male diabetic Wistar rats	[129]
Green tea alcoholic extract	↓ MDA; ↑ TAC		Streptozocin by intraperitoneal injection; 100 or 200 mg/kg green tea alcoholic extract by oral gavage for 4 weeks	Male diabetic Wistar rats	[130]

↓—decreased or inhibited concentration or activity compared to untreated group; ↑—increased concentration or activity compared to untreated group; GPX—glutathione; GSH-Px—glutathione peroxidase; SOD—superoxide dismutase; CAT—catalase; GSH—reduced glutathione; TAS—total antioxidant status; TAC—total antioxidant capacity; MDA—malondialdehyde; LPO—lipid peroxidation; TNF-α—tumor necrosis factor α; CRP—C-reactive protein; IL-6, IL-10—interleukins; NF-κB—nuclear transcription factor.

## Data Availability

Not applicable.

## References

[1] Egan A.M., Dinneen S.F. (2019). What is diabetes?. Medicine.

[2] Thant T.M., Aminah N.S., Kristanti A.N., Ramadhan R., Aung H.T., Takaya Y. (2019). Antidiabetes and Antioxidant agents from *Clausena excavata* root as medicinal plant of Myanmar. Open Chem..

[3] Tan S.Y., Mei Wong J.L., Sim Y.J., Wong S.S., Mohamed Elhassan S.A., Tan S.H., Ling Lim G.P., Rong Tay N.W., Annan N.C., Bhattamisra S.K. (2019). Type 1 and 2 diabetes mellitus: A review on current treatment approach and gene therapy as potential intervention. Diabetes Metab. Syndr..

[4] Bajaj S., Khan A. (2012). Mini Review Antioxidants and diabetes. Indian J. Endocrinol. Metab..

[5] Winiarska-Mieczan A., Baranowska-Wójcik E., Kwiecień M., Grela E.R., Szwajgier D., Kwiatkowska K., Kiczorowska B. (2020). The role of dietary antioxidants in the pathogenesis of neurodegenerative diseases and their impact on cerebral oxidoreductive balance. Nutrients.

[6] Simó R., Bañeras J., Hernández C., Rodríguez-Palomares J., Valente F., Gutierrez L., González-Alujas T., Ferreira I., Aguadé-Bruix S., Montaner J. (2019). Diabetic retinopathy as an independent predictor of subclinical cardiovascular disease: Baseline results of the PRECISED study. BMJ Open Diabetes Res. Care.

[7] Burrack A.L., Martinov T., Fife B.T. (2017). T cell-mediated beta cell destruction: Autoimmunity and alloimmunity in the context of type 1 diabetes. Front. Endocrinol..

[8] Forouhi N.G., Wareham N.J. (2014). The EPIC-InterAct Study: A Study of the Interplay between Genetic and Lifestyle Behavioral Factors on the Risk of Type 2 Diabetes in European Populations. Curr. Nutr. Rep..

[9] Galicia-Garcia U., Benito-Vicente A., Jebari S., Larrea-Sebal A., Siddiqi H., Uribe K.B., Ostolaza H., Martín C. (2020). Pathophysiology of Type 2 Diabetes Mellitus. Int. J. Mol. Sci..

[10] Nishimura A., Matsumura K., Kikuno S., Nagasawa K., Okubo M., Mori Y., Kobayashi T. (2019). Slowly progressive type 1 diabetes mellitus: Current knowledge and future perspectives. Diabetes Metab. Syndr. Obes..

[11] Pieralice S., Pozzilli P. (2018). Latent autoimmune diabetes in adults: A review on clinical implications and management. Diabetes Metab. J..

[12] Maddaloni E., Lessan N., Al Tikriti A., Buzzetti R., Pozzilli P., Barakat M.T. (2015). Latent autoimmune diabetes in adults in the United Arab Emirates: Clinical features and factors related to insulin-requirement. PLoS ONE.

[13] Fadiga L., Saraiva J., Catarino D., Frade J., Melo M., Paiva I. (2020). Adult-onset autoimmune diabetes: Comparative analysis of classical and latent presentation. Diabetol. Metab. Syndr..

[14] Yakoob A.T., Tajuddin N.B., Hussain M.I.M., Mathew S., Govindaraju A., Qadri I. (2016). Antioxidant and hypoglycemic activities of *Clausena anisata* (Wild.) Hook F. Ex Benth. Root Mediated Synthesized Silver Nanoparticles. Pharmacogn. J..

[15] Khan H., Sureda A., Belwal T., Çetinkaya S., Süntar İ., Tejada S., Devkota H.P., Ullah H., Aschner M. (2019). Polyphenols in the treatment of autoimmune diseases. Autoimmun. Rev..

[16] Hicks A. (2009). Current status and future development of global tea production and tea products. AU J. Technol..

[17] Winiarska-Mieczan A. (2018). Protective effect of tea against lead and cadmium-induced oxidative stress—A review. Biometals.

[18] Bharadwaz A., Bhattacharjee C. (2012). Extraction of polyphenols from dried tea leaves. J. Sci. Eng. Res..

[19] Winiarska-Mieczan A. (2015). The potential protective effect of green, black, red and white tea infusions against adverse effect of cadmium and lead during chronic exposure—A rat model study. Regul. Toxicol. Pharmacol..

[20] Sharma R.B., Alonso L.C. (2014). Lipotoxicity in the pancreatic beta cell: Not just survival and function, but proliferation as well?. Curr. Diabetes Rep..

[21] Bugya Z., Prechl J., Szénási T., Nemes É., Bácsi A., Koncz G. (2021). Multiple Levels of Immunological Memory and Their Association with Vaccination. Vaccines.

[22] Homsak E. (2014). Diabetes as autoimmune disease—Diabetes type I. Biochem. Med..

[23] Skrypnik D., Skrypnik K., Suliburska J., Bogdański P., Pupek-Musialik D. (2013). Dietotherapy of selected metabolic diseases. Forum Zaburzeń Metab..

[24] Xie Z., Chang C., Zhou Z. (2014). Molecular Mechanisms in Autoimmune Type 1 Diabetes: A Critical Review. Clin. Rev. Allergy Immunol..

[25] Tam A.A., Ozdemir D., Bestepe N., Dellal F.D., Bilginer M.C., Faki S., Bicer C., Ersoy R., Cakir B. (2021). Low rate of latent autoimmune diabetes in adults (LADA) in patients followed for type 2 diabetes: A single center’s experience in Turkey. Arch. Endocrinol. Metab..

[26] Carlsson S. (2019). Etiology and Pathogenesis of Latent Autoimmune Diabetes in Adults (LADA) Compared to Type 2 Diabetes. Front. Physiol..

[27] Noble J.A., Valdes A.M. (2011). Genetics of the HLA Region in the Prediction of Type 1 Diabetes. Curr. Diabetes.

[28] Noble J.A. (2015). Immunogenetics of type 1 diabetes: A comprehensive review. J. Autoimmun..

[29] Sparks A.E., Chen C., Breslin M.B., Lan M.S. (2016). Functional Domains of Autoimmune Regulator (AIRE) Modulate INS-VNTR Transcription in Human Thymic Epithelial Cells. J. Biol. Chem..

[30] Mourad D., Azar N.S., Eid A., Azar S.T. (2021). Immune Checkpoint Inhibitor-Induced Diabetes Mellitus: Potential Role of T Cells in the Underlying Mechanism. Int. J. Mol. Sci..

[31] Okruszko A., Szepietowska B., Wawrusiewicz-Kurylonek N., Górska M., Krętowski A., Szelachowska M. (2012). HLA-DR, HLA-DQB1 and PTPN22 gene polymorphism: Association with age at onset for autoimmune diabetes. Arch. Med. Sci..

[32] Howson J.M., Rosinger S., Smyth D.J., Boehm B.O., Todd J.A., ADBW-END Study Group (2011). Genetic analysis of adult-onset autoimmune diabetes. Diabetes.

[33] Qi X., Wang J., Xu Z., Keller S.J.L., Xu W. (2014). Relationship of CTLA-4 gene to latent autoimmune diabetes in adults and Type 2 diabetes: A population-based case-control study. Diabetes Manag..

[34] Giridharan S., Srinivasan M. (2018). Mechanisms of NF-κB p65 and strategies for therapeutic manipulation. J. Inflamm. Res..

[35] Chen Y., Zhou Z., Min W. (2018). Mitochondria, Oxidative Stress and Innate Immunity. Front. Physiol..

[36] Piccinni M.P., Lombardelli L., Logiodice F., Kullolli O., Parronchi P., Romagnani S. (2016). How pregnancy can affect autoimmune diseases progression?. Clin. Mol. Allergy.

[37] Ciortan L., Macarie R.D., Cecoltan S., Vadana M., Tucureanu M.M., Mihaila A.C., Droc I., Butoi E., Manduteanu I. (2020). Chronic High Glucose Concentration Induces Inflammatory and Remodeling Changes in Valvular Endothelial Cells and Valvular Interstitial Cells in a Gelatin Methacrylate 3D Model of the Human Aortic Valve. Polymers.

[38] Freemerman A.J., Johnson A.R., Sacks G.N., Milner J.J., Kirk E.L., Troester M.A., Macintyre A.N., Goraksha-Hicks P., Rathmell J.C., Makowski L. (2014). Metabolic reprogramming of macrophages: Glucose transporter 1 (GLUT1)-mediated glucose metabolism drives a proinflammatory phenotype. J. Biol. Chem..

[39] Andrisse S., Koehler R.M., Chen J.E., Patel G.D., Vallurupalli V.R., Ratliff B.A., Warren D.E., Fisher J.S. (2014). Role of GLUT1 in regulation of reactive oxygen species. Redox Biol..

[40] Clyne A.M. (2021). Endothelial response to glucose: Dysfunction, metabolism, and transport. Biochem. Soc. Trans..

[41] Klimontov V.V., Saik O.V., Korbut A.I. (2021). Glucose Variability: How Does It Work?. Int. J. Mol. Sci..

[42] Wu J., Jin Z., Zheng H., Yan L.J. (2016). Sources and implications of NADH/NAD(+) redox imbalance in diabetes and its complications. Diabetes Metab. Syndr. Obes. Targets Ther..

[43] Yan L.J. (2014). Pathogenesis of chronic hyperglycemia: From reductive stress to oxidative stress. J. Diabetes Res..

[44] Vinogradov V., Grivennikova G. (2016). Oxidation of NADH and ROS production by respiratory complex I. Biochim. Biophys. Acta Bioenergy.

[45] Kumar Rajendran N., George B.P., Chandran R., Tynga I.M., Houreld N., Abrahamse H. (2019). The Influence of Light on Reactive Oxygen Species and NF-κB in Disease Progression. Antioxidants.

[46] Iacobini C., Vitale M., Pesce C., Pugliese G., Menini S. (2021). Diabetic Complications and Oxidative Stress: A 20-Year Voyage Back in Time and Back to the Future. Antioxidants.

[47] Kleniewska P., Michalska M., Gorąca A. (2013). Influence of NADPH oxidase inhibition on oxidative stress parameters in rat hearts. Pharm. Rep..

[48] Quijano C., Trujillo M., Castro L., Trostchansky A. (2016). Interplay between oxidant species and energy metabolism. Redox Biol..

[49] Ullah A., Khan A., Khan I. (2016). Diabetes mellitus and oxidative stress—A concise review. Saudi Pharm. J..

[50] Pieme C.A., Tatangmo J.A., Simo G., Biapa Nya P.C., Ama Moor V.J., Moukette Moukette B., Tankeu Nzufo F., Njinkio Nono B.L., Sobngwi E. (2017). Relationship between hyperglycemia, antioxidant capacity and some enzymatic and non-enzymatic antioxidants in African patients with type 2 diabetes. BMC Res. Notes.

[51] Kaczmarczyk-Sedlak I., Folwarczna J., Sedlak L., Zych M., Wojnar W., Szumińska I., Wyględowska-Promieńska D., Mrukwa-Kominek E. (2019). Effect of caffeine on biomarkers of oxidative stress in lenses of rats with streptozotocin-induced diabetes. Arch. Med. Sci..

[52] Forrester S.J., Kikuchi D.S., Hernandes M.S., Xu Q., Griendling K.K. (2018). Reactive Oxygen Species in Metabolic and Inflammatory Signaling. Circ. Res..

[53] Volpe C.M.O., Villar-Delfino P.H., Dos Anjos P.M.F., Nogueira-Machado J.A. (2018). Cellular death, reactive oxygen species (ROS) and diabetic complications. Cell Death Dis..

[54] Babizhayev M.A., Strokov I.A., Nosikov V.V., Savel’yeva E.L., Sitnikov V.F., Yegorov Y.E., Lankin V.Z. (2015). The role of oxidative stress in diabetic neuropathy: Generation of free radical species in the glycation reaction and gene polymorphisms encoding antioxidant enzymes to genetic susceptibility to diabetic neuropathy in population of type I diabetic patients. Cell Biochem. Biophys..

[55] LaRocca T.-J., Sosunov S.-A., Shakerley N.-L., Ten V.-S., Ratner A.-J. (2016). Hyper-glycemic conditions prime cells for RIP1-dependent necroptosis. J. Biol. Chem..

[56] Rosa M.-D., Distefano G., Gagliano C., Rusciano D., Malaguarnera L. (2016). Autophagy in diabetic retinopathy. Curr. Neuropharmacol..

[57] Kwon D.H., Cha H.J., Lee H., Hong S.H., Park C., Park S.H., Kim G.Y., Kim S., Kim H.S., Hwang H.J. (2019). Protective Effect of Glutathione against Oxidative Stress-induced Cytotoxicity in RAW 264.7 Macrophages through Activating the Nuclear Factor Erythroid 2-Related Factor-2/Heme Oxygenase-1 Pathway. Antioxidants.

[58] Bhatti J.S., Bhatti G.K., Reddy P.H. (2017). Mitochondrial dysfunction and oxidative stress in metabolic disorders—A step towards mitochondria based therapeutic strategies. Biochim. Biophys. Acta Mol. Basis Dis..

[59] Delierneux C., Kouba S., Shanmughapriya S., Potier-Cartereau M., Trebak M., Hempel N. (2020). Mitochondrial Calcium Regulation of Redox Signaling. Cancer Cells.

[60] Ulrich K., Jakob U. (2019). The role of thiols in antioxidant systems. Free Radic. Biol. Med..

[61] Matough F.A., Budin S.B., Hamid Z.A., Alwahaibi N., Mohamed J. (2012). The role of oxidative stress and antioxidants in diabetic complications. Sultan Qaboos Univ. Med. J..

[62] Ayala A., Muñoz M.F., Argüelles S. (2014). Lipid peroxidation: Production, metabolism, and signaling mechanisms of malondialdehyde and 4-hydroxy-2-nonenal. Oxid. Med. Cell Longev..

[63] Catalá A., Díaz M. (2016). Editorial: Impact of Lipid Peroxidation on the Physiology and Pathophysiology of Cell Membranes. Front. Physiol..

[64] Cieluch A., Uruska A., Zozulinska-Ziolkiewicz D. (2020). Can We Prevent Mitochondrial Dysfunction and Diabetic Cardiomyopathy in Type 1 Diabetes Mellitus? Pathophysiology and Treatment Options. Int. J. Mol. Sci..

[65] Winiarska-Mieczan A., Mieczan T., Wójcik G. (2020). Importance of redox equilibrium in the pathogenesis of psoriasis—Impact of antioxidant-rich diet. Nutrients.

[66] Siewiera K., Łabieniec-Watała M. (2013). The role of plant polyphenols in alleviating the adverse effects of diabetes on mitochondrial homeostasis. Post Fitoter..

[67] Hua Y.Y., Zhang Y., Gong W.W., Ding Y., Shen J.R., Li H., Chen Y., Meng G.L. (2020). Dihydromyricetin improves endothelial dysfunction in diabetic mice via oxidative stress inhibition in a SIRT3-dependent manner. Int. J. Mol. Sci..

[68] Abdel-Moneim A., El-Senousy W.M., Abdel-Latif M., Khalil R.G. (2018). Association between antioxidant enzyme activities and Enterovirus-infected type 1 diabetic children. Med. Princ. Pract..

[69] Varvarovská J., Racek J., Stozický F., Soucek J., Trefil L., Pomahacová R. (2003). Parameters of oxidative stress in children with Type 1 diabetes mellitus and their relatives. J. Diabetes Complicat..

[70] Varvarovská J., Racek J., Stetina R., Sýkora J., Pomahacová R., Rusavý Z., Lacigová S., Trefil L., Siala K., Stozický F. (2004). Aspects of oxidative stress in children with type 1 diabetes mellitus. Biomed. Pharmacother..

[71] Jain S.K., McVie R., Bocchini J.A. (2006). Hyperketonemia (ketosis), oxidative stress and type 1 diabetes. Pathophysiology.

[72] Kitabchi A.E., Stentz F.B., Umpierrez G.E. (2004). Diabetic ketoacidosis induces in vivo activation of human T-lymphocytes. Biochem. Biophys. Res. Commun..

[73] Hodgkinson A.D., Bartlett T., Oates P.J., Millward B.A., Demaine A.G. (2003). The response of antioxidant genes to hyperglycemia is abnormal in patients with type 1 diabetes and diabetic nephropathy. Diabetes.

[74] Jorns A., Arndt T., Zu Vilsendorf A.M. (2014). Islet infiltration, cytokine expression and beta cell Heath in the NOD Mouse, BB rat, Komeda rat, LEW.1AR1-iddm rat and humans with type 1 diabetes. Diabetologia.

[75] Nocoń-Bohusz J., Noczyńska A. (2016). Evaluation the concentration of selected markers of the atherosclerosis process in children with diabetes type 1. Pediatr. Endocrinol..

[76] Sproston N.R., Ashworth J.J. (2018). Role of C-Reactive Protein at Sites of Inflammation and Infection. Front. Immunol..

[77] Francés D.E., Ingaramo P.I., Ronco M.T., Carnovale C.E. (2013). Diabetes, an inflammatory process: Oxidative Stress and TNF-alpha involved in hepatic complication. J. Biomed. Sci. Eng..

[78] Watters O., O’Connor J. (2011). A role for Tumor Necrosis Factor in Ischemia and Ischemic Preconditioning. J. Neuroinflamm..

[79] Dogan Y., Akarsu S., Ustundag B., Yilmaz E., Gurgoze M.K. (2006). Serum IL-1 β, IL-2, and IL-6 in insulin-dependent diabetic children. Mediat. Inflamm..

[80] Popovic D., Lalic K., Jotic A., Milicic T., Bogdanovic J., Dordevic M., Stankovic S., Jeremic V., Lalic N.M. (2019). The Inflammatory and Hemostatic Cardiovascular Risk Markers During Acute Hyperglycemic Crisis in Type 1 and Type 2 Diabetes. J. Med. Biochem..

[81] Cardoso J.F., Domingueti C.P.B., Gomes K., Fernandes A.P. (2017). Evaluation of cytokines in type 1 diabetes patients with and without retinopathy. J. Bras. Patol. Med. Lab..

[82] Wang Y., Qi W., Song G., Pang S., Peng Z., Li Y., Wang P. (2020). High-Fructose Diet Increases Inflammatory Cytokines and Alters Gut Microbiota Composition in Rats. Mediat. Inflamm..

[83] Zhang L., Gui S., Wang J., Chen Q., Zeng J., Liu A., Chen Z., Lu X. (2019). Oral administration of green tea polyphenols (TP) improves ileal injury and intestinal flora disorder in mice with *Salmonella typhimurium* infection via resisting inflammation, enhancing antioxidant action and preserving tight junction. J. Funct. Foods.

[84] Othman Z.A., Zakaria Z., Suleiman J.B., Ghazali W., Mohamed M. (2021). Anti-Atherogenic Effects of Orlistat on Obesity-Induced Vascular Oxidative Stress Rat Model. Antioxidants.

[85] Mahdavifard S., Nakhjavani M. (2019). Effect of Glutamine on Oxidative Stress, Inflammatory, and Glycation Markers, and the Activity of Glyoxalase System in Diabetic Rats with Atherosclerosis. J. Maz. Univ. Med. Sci..

[86] Mittal M., Siddiqui M.R., Tran K., Reddy S.P., Malik A.B. (2014). Reactive oxygen species in inflammation and tissue injury. Antioxid. Redox Signal..

[87] Marshall J.S., Warrington R., Watson W., Kim H.L. (2018). An introduction to immunology and immunopathology. Allergy Asthma Clin. Immunol..

[88] Fioranelli M., Roccia M.G., Flavin D., Cota L. (2021). Regulation of Inflammatory Reaction in Health and Disease. Int. J. Mol. Sci..

[89] Belhiba O., Aadam Z., Jeddane L., Saile R., Salih A.L.J.H., Bousfiha A.A., Jennane F. (2020). Research of anti-GAD and anti-IA2 autoantibodies by ELISA test in a series of Moroccan pediatric patients with diabetes type 1. Afr. Health Sci..

[90] Kikkas I., Mallone R., Larger E., Volland H., Morel N. (2014). A Rapid Lateral Flow Immunoassay for the Detection of Tyrosine Phosphatase-Like Protein IA-2 Autoanti-bodies in Human Serum. PLoS ONE.

[91] Zamanfar D., Mohsen A., Monireh A., Monajati M. (2020). Prevalence of autoantibodies in type 1 diabetes mellitus pediatrics in Mazandaran, North of Iran. J. Pediatr. Endocrinol. Metab..

[92] Delitala A.P. (2019). Autoimmunity in latent autoimmune diabetes in adults. AIMS Med. Sci..

[93] Pipi E., Marketou M., Tsirogianni A. (2014). Distinct clinical and laboratory characteristics of latent autoimmune diabetes in adults in relation to type 1 and type 2 diabetes mellitus. World J. Diabetes.

[94] Szepietowska B., Wawrusiewicz-Kurylonek N., Krętowski A., Górska M., Szelachowska M. (2016). Endocrine autoimmunity in patients with Latent Autoimmune Diabetes in Adults (LADA)—Association with HLA genotype. Endokrynol. Pol..

[95] Unanue E.R., Ferris S.T., Carrero J.A. (2016). The role of islet antigen presenting cells and the presentation of insulin in the initiation of autoimmune diabetes in the NOD mouse. Immunol. Rev..

[96] Li Y., Sun F., Yue T.T., Wang F.X., Yang C.L., Luo J.H., Rong S.J., Xiong F., Zhang S., Wang C.Y. (2021). Revisiting the Antigen-Presenting Function of β Cells in T1D Pathogenesis. Front. Immunol..

[97] Hänninen A., Harrison L.C. (2000). T cels as mediators of mucosal tolerance: The autoimmune diabetes model. Immunol. Rev..

[98] Simos Y.V., Verginadis I.I., Toliopoulos I.K., Velalopoulou A.P., Karagounis I.V., Karkabounas S.C., Evangelou A.M. (2012). Effects of catechin and epicatechin on superoxide dismutase and glutathione peroxidase activity, in vivo. Redox Rep..

[99] Amorati R., Baschieri A., Cowden A., Valgimigli L. (2017). The antioxidant activity of quercetin in water solution. Biomimetics.

[100] Shannon E., Jaiswal A.K., Abu-Ghannam N. (2018). Polyphenolic content and antioxidant capacity of white, green, black, and herbal teas: A kinetic study. Food Res..

[101] Wołosiak R., Mazurkiewicz M., Drużyńska B., Worobiej E. (2008). Antioxidant activity of the selected green teas. Żywn Nauka Technol. Jakość.

[102] Korir M.W., Wachira F.N., Wanyoko J.K., Ngure R.M., Khalid R. (2014). The fortification of tea with sweeteners and milk and its effect on in vitro antioxidant potential of tea product and glutathione levels in an animal model. Food Chem..

[103] Winiarska-Mieczan A. (2013). Protective effect of tannic acid on the brain of adult rats exposed to cadmium and lead. Environ. Toxicol. Pharmacol..

[104] Ibrahim N.K. (2013). Possible protective effect of kombucha tea ferment on cadmium chloride induced liver and kidney damage in irradiated rats. Int. J. Biol. Life Sci..

[105] El-Beltagy M.A., Saleh S.Y., El-Ghannam A.E.R., Ibrahim I.A. (2015). Protective effect of green tea extract on heavy metals-induced oxidative testicular damage in rats. Indian J. Appl. Res..

[106] Nna V.U., Usman U.Z., Ofulet E.O., Owu D.U. (2017). Quercetin exerts preventive, ameliorative and prophylactic effects on cadmium chloride-induced oxidative stress in the uterus and ovaries of female Wistar rats. Food Chem. Toxicol..

[107] Zargar S., Siddiqi N.J., Al Daihan S.K., Wani T. (2015). Protective effects of quercetin on cadmium fluoride induced oxidative stress at different intervals of time in mouse liver. Acta Biochim. Pol..

[108] Yi R., Wang R., Sun P., Zhao X. (2015). Antioxidant-mediated preventative effect of Dragon-pearl tea crude polyphenol extract on reserpine-induced gastric ulcers. Exp. Med..

[109] Ahmed N.A., Radwan N.M., Aboul Ezz H.S., Salama N.A. (2017). The antioxidant effect of green tea mega EGCG against electromagnetic radiation-induced oxidative stress in the hippocampus and striatum of rats. Electromagn. Biol. Med..

[110] Zhang Z.Y., Miao L.F., Qian L.L., Wang N., Qi M.M., Zhang Y.M., Dang S.P., Wu Y., Wang R.X. (2019). Molecular Mechanisms of Glucose Fluctuations on Diabetic Complications. Front. Endocrinol..

[111] Jiang L., Tang C., Rao J., Xue Q., Wu H., Wu D., Zhang A., Chen L., Shen Z., Lei L. (2017). Systematic identification of the druggable interactions between human protein kinases and naturally occurring compounds in endometriosis. Comput. Biol. Chem..

[112] Du G.J., Zhang Z., Wen X.D., Yu C., Calway T., Yuan C.S., Wang C.Z. (2012). Epigallocatechin Gallate (EGCG) is the most effective cancer chemopreventive polyphenol in green tea. Nutrients.

[113] Fatima M., Kesharwani R.K., Misra K., Rizvi S.I. (2013). Protective effect of theaflavin on erythrocytes subjected to in vitro oxidative stress. Biochem. Res. Int..

[114] Prince P.D., Lanzi C.R., Toblli J.E., Elesgaray R., Oteiza P.I., Fraga C.G., Galleano M. (2016). Dietary (-)-epicatechin mitigates oxidative stress, NO metabolism alterations, and inflammation in renal cortex from fructose-fed rats. Free Radic. Biol. Med..

[115] Litterio M.C., Vazquez Prieto M.A., Adamo A.M., Elesgaray R., Oteiza P.I., Galleano M., Fraga C.G. (2015). (-)-Epicatechin reduces blood pressure increase in high-fructose-fed rats: Effects on the determinants of nitric oxide bioavailability. J. Nutr. Biochem..

[116] Calabró V., Piotrkowski B., Fischerman L., Prieto M.A.V., Galleano M., Fraga C.G. (2016). Modifications in nitric oxide and superoxide anion metabolism induced by fructose overload in rat heart are prevented by (−)-epicatechin. Food Funct..

[117] Prince P.D., Lanzi C.R., Fraga C.G., Galleano M. (2019). Dietary (−)-epicatechin affects NF-κB activation and NADPH oxidases in the kidney cortex of high-fructose-fed rats. Food Funct..

[118] Thichanpiang P., Wongprasert K. (2015). Green tea polyphenol epigallocatechin-3-gallate attenuates TNF-α-induced intercellular adhesion molecule-1 expression and monocyte adhesion to retinal pigment epithelial cells. Am. J. Chin. Med..

[119] Gothandam K., Ganesan V.S., Ayyasamy T., Ramalingam S. (2019). Antioxidant potential of theaflavin ameliorates the activities of key enzymes of glucose metabolism in high fat diet and streptozotocin—Induced diabetic rats. Redox Rep..

[120] Bulboaca A.E., Boarescu P.M., Porfire A.S., Dogaru G., Barbalata C., Valeanu M., Munteanu C., Râjnoveanu R.M., Nicula C.A., Stanescu I.C. (2020). The Effect of Nano-Epigallocatechin-Gallate on Oxidative Stress and Matrix Metalloproteinases in Experimental Diabetes Mellitus. Antioxidants.

[121] Leu J.G., Lin C.Y., Jian J.H., Shih C.Y., Liang Y.J. (2013). Epigallocatechin-3-gallate combined with alpha lipoic acid attenuates high glucose-induced receptor for advanced glycation end products (RAGE) expression in human embryonic kidney cells. An. Acad. Bras. Cienc..

[122] Samarghandian S., Azimi-Nezhad M., Farkhondeh T. (2017). Catechin Treatment Ameliorates Diabetes and Its Complications in Streptozotocin-Induced Diabetic Rats. Dose Response.

[123] Mota M.A., Landim J.S., Targino T.S., Silva S.F., Silva S.L., Pereira M.R. (2015). Evaluation of the anti-inflammatory and analgesic effects of green tea (*Camellia sinensis*) in mice. Acta Cir. Bras..

[124] Szulińska M., Stępień M., Kręgielska-Narożna M., Suliburska J., Skrypnik D., Bąk-Sosnowska M., Kujawska-Łuczak M., Grzymisławska M., Bogdański P. (2017). Effects of green tea supplementation on inflammation markers, antioxidant status and blood pressure in NaCl-induced hypertensive rat model. Food Nutr. Res..

[125] Abolfathi A.A., Mohajeri D., Rezaie A., Nazeri M. (2012). Protective Effects of Green Tea Extract against Hepatic Tissue Injury in Streptozotocin-Induced Diabetic Rats. Evid.-Based Complement. Altern. Med..

[126] Al-Shiekh A.A.M., Al-Shati A.A., Sarhan M.A.A. (2014). Effect of White Tea Extract on Antioxidant Enzyme Activities of Streptozotocin—Induced Diabetic Rats. Egypt. Acad. J. Biolog. Sci..

[127] Sharifzadeh M., Ranjbar A., Hosseini A., Khanavi M. (2017). The Effect of Green Tea Extract on Oxidative Stress and Spatial Learning in Streptozotocin-diabetic Rats. Iran. J. Pharm. Sci..

[128] Rohman M.S., Lukitasari M., Nugroho D.A., Ramadhiani R., Widodo N., Kusumastuty I., Nugrahini N.I.P. (2021). Decaffeinated light-roasted green coffee and green tea extract combination improved metabolic parameters and modulated inflammatory genes in metabolic syndrome rats. F1000Research.

[129] Gennaro G., Claudino M., Cestari T.M., Ceolin D., Germino P., Garlet G.P., De Assis G.F. (2015). Green tea modulates cytokine expression in the periodontium and attenuates alveolar bone resorption in type 1 diabetic rats. PLoS ONE.

[130] Haidari F., Omidian K., Rafiei H., Zarei M., Mohamad Shahi M. (2013). Green Tea (*Camellia sinensis*) Supplementation to Diabetic Rats Improves Serum and Hepatic Oxidative Stress Markers. Iran. J. Pharm. Sci..

[131] Carito V., Ciafrè S., Tarani L., Ceccanti M., Natella F., Iannitelli A., Tirassa P., Chaldakov G.N., Ceccanti M., Boccardo C. (2015). TNF-α and IL-10 modulation induced by polyphenols extracted by olive pomace in a mouse model of paw inflammation. Ann. Ist. Super. Sanita.

[132] Yahfoufi N., Alsadi N., Jambi M., Matar C. (2018). The Immunomodulatory and Anti-Inflammatory Role of Polyphenols. Nutrients.

[133] Saleh H.A., Yousef M.H., Abdelnaser A. (2021). The Anti-Inflammatory Properties of Phytochemicals and Their Effects on Epigenetic Mechanisms Involved in TLR4/NF-κB-Mediated Inflammation. Front. Immunol..

[134] Owczarek K., Fichna J., Lewandowska U. (2017). Anti-inflammatory activity of polyphenolic compounds. Post Fitoter..

[135] Wang S., Li Z., Ma Y., Liu Y., Lin C.C., Li S., Zhan J., Ho C.T. (2021). Immunomodulatory Effects of Green Tea Polyphenols. Molecules.

[136] Zhou T., Zhu M., Liang Z. (2018). (−)-Epigallocatechin-3-gallate modulates peripheral immunity in the MPTP-induced mouse model of Parkinson’s disease. Mol. Med. Rep..

[137] Li J., Yip Y.W.Y., Ren J., Hui W.K., He J.N., Yu Q.X., Chu K.O., Ng T.K., Chan S.O., Pang C.P. (2019). Green tea catechins alleviate autoimmune symptoms and visual impairment in a murine model for human chronic intraocular inflammation by inhibiting Th17-associated pro-inflammatory gene expression. Sci. Rep..

[138] Cao Y., Wang D., Wang X., Zhang J., Shan Z., Teng W. (2013). (-)-Epigallocatechin gallate inhibits TNF-α-induced PAI-1 production in vascular endothelial cells. J. Cardiovasc. Pharmacol..

[139] Bogdański P., Szulińska M., Dytfeld J., Pupek-Musialik D. (2012). Evaluation of plasminogen activator inhibitor 1 concentration in patients with simple obesity. Endocrinol. Obes. Metab. Disord..

[140] Riegsecker S., Wiczynski D., Kaplan M.J., Ahmed S. (2013). Potential benefits of green tea polyphenol EGCG in the prevention and treatment of vascular inflammation in rheumatoid arthritis. Life Sci..

[141] Wang Z.M., Gao W., Wang H., Zhao D., Nie Z.L., Shi J.Q., Zhao S., Lu X., Wang L.S., Yang Z.J. (2014). Green tea polyphenol epigallocatechin-3-gallate inhibits TNF-α-induced production of monocyte chemoattractant protein-1 in human umbilical vein endothelial cells. Cell Physiol. Biochem..

[142] Li M., Liu J.T., Pang X.M., Han C.J., Mao J.J. (2012). Epigallocatechin-3-gallate inhibits angiotensin II and interleukin-6-induced C-reactive protein production in macrophages. Pharm. Rep..

[143] Bagheri R., Rashidlamir A., Ashtary-Larky D., Wong A., Alipour M., Motevalli M.S., Chebbi A., Laher I., Zouhal H. (2020). Does green tea extract enhance the anti-inflammatory effects of exercise on fat loss?. Br. J. Clin. Pharmacol..

[144] Yilmaz R., Akoglu H., Altun B., Yildirim T., Arici M., Erdem Y. (2012). Dietary salt intake is related to inflammation and albuminuria in primary hypertensive patients. Eur. J. Clin. Nutr..

[145] Chen B.T., Li W.X., He R.R., Li Y.F., Tsoi B., Zhai Y.J., Kurihara H. (2012). Anti-inflammatory effects of a polyphenols-rich extract from tea (*Camellia sinensis*) flowers in acute and chronic mice models. Oxid. Med. Cell Longev..

[146] Chatterjee P., Chandra S., Dey P., Bhattacharya S. (2012). Evaluation of anti-inflammatory effects of green tea and black tea: A comparative in vitro study. J. Adv. Pharm. Technol. Res..

[147] Shakoor H., Feehan J., Apostolopoulos V., Platat C., Al Dhaheri A.S., Ali H.I., Ismail L.C., Bosevski M., Stojanovska L. (2021). Immunomodulatory Effects of Dietary Polyphenols. Nutrients.

[148] Ding S., Jiang H., Fang J. (2018). Regulation of Immune Function by Polyphenols. J. Immunol. Res..

[149] Min S.Y., Yan M., Kim S.B., Ravikumar S., Kwon S.R., Vanarsa K., Kim H.Y., Davis L.S., Mohan C. (2015). Green Tea Epigallocatechin-3-Gallate Suppresses Autoimmune Arthritis Through Indoleamine-2,3-Dioxygenase Expressing Dendritic Cells and the Nuclear Factor, Erythroid 2-Like 2 Antioxidant Pathway. J. Inflamm..

[150] Wu D., Wang J. (2011). The ability of green tea to alleviate autoimmune diseases: Fact or fiction?. Expert Rev. Clin. Immunol..

[151] Huang S.C., Kao Y.H., Shih S.F., Tsai M.C., Lin C.S., Chen L.W., Chuang Y.P., Tsui P.F., Ho L.J., Lai J.H. (2021). Epigallocatechin-3-gallate exhibits immunomodulatory effects in human primary T cells. Biochem. Biophys. Res. Commun..

[152] Deng Q., Xu J., Yu B., He J., Zhang K., Ding X., Chen D. (2010). Effect of dietary tea polyphenols on growth performance and cell mediated immune response of post-weaning piglets under oxidative stress. Arch. Anim. Nutr..

[153] Wang Z., Sun B., Zhu F. (2018). Epigallocatechin-3-gallate protects Kuruma shrimp *Marsupeneaus japonicus* from white spot syndrome virus and *Vibrio alginolyticus*. Fish. Shellfish Immun..

[154] Meng J.M., Cao S.Y., Wei X.L., Gan R.Y., Wang Y.F., Cai S.X., Xu X.Y., Zhang P.Z., Li H.B. (2019). Effects and Mechanisms of Tea for the Prevention and Management of Diabetes Mellitus and Diabetic Complications: An Updated Review. Antioxidants.

[155] Fu Q.Y., Li Q.S., Lin X.M., Qiao R.Y., Yang R., Li X.M., Dong Z.B., Xiang L.P., Zheng X.Q., Lu J.L. (2017). Antidiabetic Effects of Tea. Molecules.

[156] Sharma V., Gupta A.K., Walia A. (2019). Effect of Green Tea on Diabetes Mellitus. ASNH.

[157] Li B., Fu L., Kojima R., Yamamoto A., Uemo T., Matsui T. (2021). Theaflavins prevent the onset of diabetes through ameliorating glucose tolerance mediated by promoted incretin secretion in spontaneous diabetic Torii rats. J. Funct. Foods.

[158] Yang K., Hashemi Z., Han W., Jin A., Yang H., Ozga J., Li L., Chan C.B. (2015). Hydrolysis enhances bioavailability of proanthocyanidin-derived metabolites and improves β-cell function in glucose intolerant rats. J. Nutr. Biochem..

[159] Suzuki T., Pervin M., Goto S., Isemura M., Nakamura Y. (2016). Beneficial effects of tea and the green tea catechin epigallocatechin-3-gallate on obesity. Molecules.

[160] Yang K., Chan C. (2018). Epicatechin potentiation of glucose-stimulated insulin secretion in INS-1 cells is not dependent on its antioxidant activity. Acta Pharm. Sin..

[161] Komorita Y., Iwase M., Fujii H., Ohkuma T., Ide H., Jodai-Kitamura T., Yoshinari M., Oku Y., Higashi T., Nakamura U. (2020). Additive effects of green tea and coffee on all-cause mortality in patients with type 2 diabetes mellitus: The Fukuoka Diabetes Registry. BMJ Open Diabetes Res. Care.

[162] Ninomiya T., Kanzaki N., Hirakawa Y., Yoshinari M., Higashioka M., Honda T., Shibata M., Sakata S., Yoshida D., Teramoto T. (2019). Serum Ethylamine Levels as an Indicator of l-Theanine Consumption and the Risk of Type 2 Diabetes in a General Japanese Population: The Hisayama Study. Diabetes Care.

[163] Adi P.J., Burra S.P., Vataparti A.R., Matcha B. (2016). Calcium, zinc and vitamin E ameliorate cadmium-induced renal oxidative damage in albino Wistar rats. Toxicol. Rep..

[164] Sarkar D., Bose S.K., Chakraborty T., Roy S. (2021). Theaflavin Enriched Black Tea Extract Alleviates Diabetic Nephropathy by Suppressing Hyperglycaemia-Mediated Oxidative Stress and Inflammation in Streptozotocin-Induced Rats. Nat. Prod. J..

[165] Rusak G., Šola I., Vujčić Bok V. (2021). Matcha and Sencha green tea extracts with regard to their phenolics pattern and antioxidant and antidiabetic activity during in vitro digestion. J. Food Sci. Technol..

[166] Ramírez-Sánchez I., Rodríguez A., Moreno-Ulloa A., Ceballos G., Villarreal F. (2016). (-)-Epicatechin-induced recovery of mitochondria from simulated diabetes: Potential role of endothelial nitric oxide synthase. Diabetes Vasc. Dis. Res..

[167] Joo S.Y., Song Y.A., Park Y.L., Myung E., Chung C.Y., Park K.J., Cho S.B., Lee W.S., Kim H.S., Rew J.S. (2012). Epigallocatechin-3-gallate Inhibits LPS-Induced NF-κB and MAPK Signaling Pathways in Bone Marrow-Derived Macrophages. Gut Liver.

[168] Deng X., Hou Y., Zhou H., Li Y., Xue Z., Xue X., Huang G., Huang K., He X., Xu W. (2021). Hypolipidemic, anti-inflammatory, and anti-atherosclerotic effects of tea before and after microbial fermentation. Food Sci. Nutr..

[169] Manzel A., Muller D.N., Hafler D.A., Erdman S.E., Linker R.A., Kleinewietfeld M. (2014). Role of “Western diet” in inflammatory autoimmune diseases. Curr. Allergy Asthma Rep..

[170] Pérez-Burillo S., Navajas-Porras B., López-Maldonado A., Hinojosa-Nogueira D., Pastoriza S., Rufián-Henares J.Á. (2021). Green Tea and Its Relation to Human Gut Microbiome. Molecules.

[171] Al Bander Z., Nitert M.D., Mousa A., Naderpoor N. (2020). The Gut Microbiota and Inflammation: An Overview. Int. J. Environ. Res. Public Health.

[172] Samriz O., Mizrahi H., Werbner M., Shoenfeld Y., Avni O., Koren O. (2016). Microbiota at the crossroads of autoimmunity. Autoimmun Rev..

[173] De Luca F., Shoenfeld Y. (2019). The microbiome in autoimmune diseases. Clin. Exp. Immunol..

[174] Dias R., Bergamo P., Maurano F., Aufiero V.R., Luongo D., Mazzarella G., Bessa-Pereira C., Pérez-Gregorio M., Rossi M., Freitas V. (2021). First morphological-level insights into the efficiency of green tea catechins and grape seed procyanidins on a transgenic mouse model of celiac disease enteropathy. Food Funct..

[175] Westerlind H., Palmqvist I., Saevarsdottir S., Alfredsson Lars Klareskog L., Di Giuseppe D. (2021). Is tea consumption associated with reduction of risk of rheumatoid arthritis? A Swedish case-control study. Arthritis Res. Ther..

[176] Dias R., Brás N., Fernandes I., Pérez-Gregorio M., Mateus N., Freitas V. (2018). Molecular insights on the interaction and preventive potential of epigallocatechin-3-gallate in Celiac Disease. Int. J. Biol. Macromol..

[177] Huynh N.B. (2017). The Immunological Benefits of Green Tea (*Camellia sinensis*). Int. J. Biol..

[178] Berná G., Oliveras-López M.J., Jurado-Ruíz E., Tejedo J., Bedoya F., Soria B., Martín F. (2014). Nutrigenetics and nutrigenomics insights into diabetes etiopathogenesis. Nutrients.

[179] Akil A.A., Jerman L.F., Yassin E., Padmajeya S.S., Al-Kurbi A., Fakhro K.A. (2020). Reading between the (Genetic) Lines: How Epigenetics is Unlocking Novel Therapies for Type 1 Diabetes. Cells.

[180] Poczęta M., Nowak E., Bieg D., Bednarek I. (2018). Epigenetic modifications and gene expression in cancerogenesis. Ann. Acad. Med. Silesiensis.

[181] Al Theyab A., Almutairi T., Al-Suwaidi A.M., Bendriss G., McVeigh C., Chaari A. (2020). Epigenetic Effects of Gut Metabolites: Exploring the Path of Dietary Prevention of Type 1 Diabetes. Front. Nutr..

[182] Gallo E., Maggini V., Berardi M., Pugi A., Notaro R., Talini G., Vannozzi G., Bagnoli S., Forte P., Mugelli A. (2013). Is green tea a potential trigger for autoimmune hepatitis?. Phytomedicine.

[183] Yang E.J., Lee J., Lee S.Y., Kim E.K., Moon Y.M., Jung Y.O., Cho M.L. (2014). EGCG attenuates autoimmune arthritis by inhibition of STAT3 and HIF-1α with Th17/Treg control. PLoS ONE.

[184] Rana S., Kumar S., Rathore N., Padwad Y., Bhushana S. (2016). Nutrigenomics and its Impact on Life Style Associated Metabolic Diseases. Curr. Genom..

[185] Ortsäter H., Grankvist N., Wolfram S., Kuehn N., Sjöholm A. (2012). Diet supplementation with green tea extract epigallocatechin gallate prevents progression to glucose intolerance in db/db mice. Nutr. Metab..

[186] Zhang Z., Ding Y., Dai X., Wang J., Li Y. (2011). Epigallocatechin-3-gallate protects pro-inflammatory cytokine induced injuries in insulin-producing cells through the mitochondrial pathway. Eur. J. Pharmacol..

[187] Palacio Sánchez E., Ribero Vargas M.E., Restrepo Gutiérrez J.C. (2013). Hepatotoxicity due to green tea consumption (*Camellia sinensis*): A review. Rev. Col. Gastroenterol..

[188] Reddy M.A., Kumar B.K., Boobalan G., Reddy M.K., Kumar C.S.V.S., Reddy G.A., Lakshman M. (2017). Hepatoprotective potential of green tea extract against experimental hepatotoxicity in rats. Indian J. Pharm Sci..

[189] Ali A.H.A. (2018). Hepatoprotective effect of green tea extractagainst cyclophosphamide induced liver injury in albino rats. FMAR.

[190] Esposito S., Toni G., Tascini G., Santi E., Berioli M.G., Principi N. (2019). Environmental Factors Associated with Type 1 Diabetes. Front. Endocrinol..

